# GluN2A Enables Noradrenergic Control of Prefrontal Oscillations and Cognitive Flexibility

**DOI:** 10.1523/ENEURO.0050-26.2026

**Published:** 2026-08-04

**Authors:** Hassan Hosseini, Sky Evans-Martin, Emma Bogomilsky, Kevin S. Jones

**Affiliations:** ^1^Department of Pharmacology, University of Michigan Medical School, Ann Arbor, Michigan 48109; ^2^Neuroscience Graduate Program, University of Michigan Medical School, Ann Arbor, Michigan 48109

**Keywords:** cognitive flexibility, gamma oscillations, locus ceruleus, medial prefrontal cortex, NMDA receptor GluN2A, norepinephrine

## Abstract

Cognitive flexibility—the ability to adapt behavior when contingencies change—is impaired in psychiatric disorders involving prefrontal dysfunction. The medial prefrontal cortex (mPFC) relies on noradrenergic input from the locus ceruleus (LC), yet the molecular mechanisms enabling this neuromodulatory control remain unclear. Here we show that GluN2A-containing NMDA receptors are required for LC–mPFC regulation of network dynamics and reversal learning in male mice. Optogenetic activation of LC→mPFC projections enhanced reversal learning in wild-type (WT) and heterozygous mice but not in global *Grin2a* knock-outs, whereas LC inhibition impaired performance only in WT animals. In slices, norepinephrine (NE) and LC stimulation induced gamma and high-frequency oscillations in WT mPFC that were blocked by α2-adrenergic antagonism, but these oscillatory responses were undetectable in *Grin2a* mutants. *Grin2a* mutants also exhibited increased LC axonal density and elevated NE transporter expression in the prelimbic cortex, consistent with enhanced noradrenergic clearance capacity. Together, these findings identify GluN2A as a key determinant of LC–prefrontal circuit function supporting cognitive flexibility. They suggest that functional deficits in these mutants should be interpreted within the context of compensatory structural hyperinnervation resulting from global GluN2A deficiency, which may reflect a developmental adaptation rather than acute signaling loss. Furthermore, these results promote α2-adrenergic pathways as potential entry points for restoring prefrontal network coordination.

## Significance Statement

Cognitive flexibility is often impaired in psychiatric disorders, yet how norepinephrine engages prefrontal network dynamics remains unclear. We investigate how global GluN2A deficiency affects LC–mPFC dynamics while accounting for potential systemic and developmental contributions. These findings show that global GluN2A deficiency disrupts noradrenergic induction of synchronized oscillatory activity and highlight *α*2-adrenergic pathways as a candidate therapeutic entry point for improving prefrontal network function. Importantly, our results suggest that functional deficits in global knock-out models must be interpreted within the context of compensatory structural changes in circuit architecture, such as hyperinnervation.

## Introduction

Cognitive flexibility—the capacity to adjust behavior when contingencies change—depends on the integrity of prefrontal circuits and is disrupted in many neuropsychiatric disorders (Millan et al., 2012). The medial prefrontal cortex (mPFC) receives dense noradrenergic input from the locus ceruleus (LC), which modulates excitability and helps coordinate cortical activity across behavioral states (Aston-Jones and Cohen, 2005; McBurney-Lin et al., 2022). Phasic norepinephrine (NE) release can promote gamma-frequency synchronization and support task switching, mediated in part by α2-adrenergic receptors that stabilize recurrent activity through suppression of cAMP-dependent K^+^ conductances (Ramos and Arnsten, 2007; Wang et al., 2007). Although adrenergic mechanisms supporting attention and working memory are well defined (Wang et al., 2013), how NE engages prefrontal network rhythms to support behavioral flexibility—and how this control fails in disease-relevant states—remains unclear. Dysregulated noradrenergic signaling within mPFC circuits has been implicated in cognitive inflexibility and neuropsychiatric vulnerability, motivating efforts to define how LC-derived NE recruits coordinated prefrontal dynamics.

Mutations in GRIN2A, encoding the GluN2A subunit of NMDA receptors, are linked to cognitive rigidity and psychiatric vulnerability (Singh et al., 2022). GluN2A-containing receptors govern synaptic maturation, temporal integration, and gamma-band synchrony during cognitively demanding states (Ryan et al., 2013; Hanson et al., 2020). Noradrenergic and glutamatergic signaling converge on prefrontal pyramidal neurons to regulate network precision (Xing et al., 2016), yet it is unknown whether GluN2A is required for NE to recruit synchronized activity patterns in mPFC. More broadly, LC and mPFC form a reciprocal circuit in which ascending neuromodulatory drive and descending cortical feedback jointly shape adaptive control, raising the possibility that GluN2A dysfunction destabilizes this loop.

We hypothesized that GluN2A deficiency disrupts noradrenergic regulation of mPFC network activity, leading to impaired behavioral flexibility. To test this, we combined behavioral, optogenetic, pharmacological, and electrophysiological approaches in *Grin2a* mutant mice. We assessed reversal learning, measured how LC→mPFC manipulations shape prefrontal synchronization, and examined both circuit structure and LC physiological properties. Together, these experiments test how a defined genetic risk factor is associated with altered LC–mPFC structure and function in a model where behavioral and circuit phenotypes may also reflect broader developmental consequences of global GluN2A deficiency.

While mutations in *GRIN2A* are a primary genetic risk factor for cognitive rigidity, their impact on specific neuromodulatory pathways remains poorly defined. In this study, we utilize a global *Grin2a* knock-out model to investigate how GluN2A deficiency disrupts the functional and structural integrity of the LC–mPFC circuit. Although the global nature of this deficiency means that observed behavioral deficits may reflect systemic contributions or developmental adaptations across multiple brain regions, this model is essential for identifying the compensatory structural reorganization—such as the noradrenergic hyperinnervation, we describe here—that emerges when GluN2A-dependent signaling is chronically absent. By characterizing these circuit-level failures, we aim to define the broader mechanisms through which genetic vulnerability translates into impaired prefrontal network coordination and diminished cognitive flexibility.

## Materials and Methods

### Animals

All procedures were approved by the Institutional Animal Care and Use Committee and conducted in accordance with NIH guidelines. *Grin2a* wild-type (WT), heterozygous (*Grin2a*^HET^), and homozygous knock-out (*Grin2a*^KO^) mice (Ryan et al., 2013) were group-housed under a 12 h light/dark cycle (lights on at 07:00) with food and water available *ad libitum*. Genotyping was performed by PCR from tail snips.

### Stereotaxic surgery and viral injections

Surgeries were performed on 7–8-week-old male mice anesthetized with isoflurane (4–5% for induction; 1–2% for maintenance) and secured in a stereotaxic frame (David Kopf Instruments, Model 1400). Viral injections were delivered through a pipette (10–12 µm) pulled from glass capillaries (Drummond, 3-000-203-G/X) attached to a Nanoject II microinjector (Drummond).

To optimize optogenetic manipulations across experimental preparations, distinct opsins were used: ChETA for high-fidelity somatic stimulation in behavioral assays and ChR2 for robust terminal stimulation ex vivo while maintaining a constant viral injection volume (110 nl) across cohorts. To label LC→mPFC projection neurons, mice received a unilateral injection of retrograde Cre virus (pENN.AAV.hSyn.HI.eGFP-Cre.WPRE.SV40; Addgene #105540-AAVrg, 10^12^ vg/ml) into the right mPFC (AP +1.9 mm, ML +0.3 mm, DV −1.8 mm; 110 nl). Cre-dependent opsin viruses were injected into the right LC (AP −5.4 mm, ML +0.85 mm, DV −4.0 mm; 110 nl), consisting of either an excitatory construct (pAAV-Ef1a-DIO-ChETA-EYFP; Addgene #26968-AAV9, 10^13^ GC/ml) or an inhibitory construct (pAAV-hSyn1-SIO-stGtACR2-FusionRed; Addgene #105677-AAV1, 10^12^ GC/ml).

### Optical fiber implantation

In the same surgical session, a ferrule-coupled optical fiber (200 µm core, 0.22 NA; RWD R-FOC-L200C22NA) was implanted 150 µm above the LC injection site (final DV, −3.7 mm) and secured to the skull using dental acrylic (Ortho-Jet Orthodontic Resin Self Cure 100Gm/Pk #1250630). Postoperative care included subcutaneous carprofen (5 mg/kg) administered once daily for 3 d. Mice were given 2–3 weeks for viral expression and recovery before any behavioral or electrophysiological testing.

### T-maze reversal learning task

Behavioral testing began ∼2–3 weeks after viral surgery. Mice were mildly food-restricted to 75–85% of their free-feeding weight and fasted for 10–12 h prior to each session. The reversal learning task was conducted in a custom-built T-maze (arm dimensions, 50 × 7 × 20 cm; start box, 15 × 15 cm) equipped with manually operated doors.

Mice underwent 2 d of habituation, during which they explored the maze freely for 10 min per session with all doors open and sucrose pellets available in both goal arms. This was followed by 2 d of behavioral shaping (10 trials/day), where mice were trained to retrieve a sucrose pellet (45 mg) from alternating arms. Mice that completed each trial within 1–3 min were advanced to the training phase.

During acquisition, each mouse learned to associate a fixed goal arm (left or right, counterbalanced across animals) with reward. Sessions consisted of 10 trials, and mice were considered to have reached criterion when they achieved >70% correct choices in a session.

Following acquisition, the reward location was reversed such that the previously nonrewarded arm became correct. Unilateral optogenetic stimulation of the right LC was delivered only during the second reversal. Optical stimulation was delivered through the implanted fiber, and, to avoid nonspecific suppression of global arousal, unilateral LC targeting was used (Janitzky et al., 2015). Mice received either a 25 Hz pulsed photoexcitation or 10 s constant photoinhibition while in the start box; stimulation was delivered immediately before door opening. Light (14–17 mW) was delivered through an implantable cannula (200 µm core, 0.22 NA; RWD R-FOC-L200C22NA) connected via a mating sleeve to an optical patch cable, and laser power was adjusted to achieve the stated power at the cannula output (the implanted cannula's NA defines the emission cone in the tissue).

### Whole-cell patch–clamp recordings

Acute 300 µm coronal slices of mPFC/LC were prepared from adult WT, *Grin2a*^KO^, or *Grin2a*^HET^ mice (10–12 weeks) using an NMDG-HEPES protective recovery protocol (Ting et al., 2018). Brains were rapidly extracted in ice-cold NMDG cutting solution, incubated at 32°C for 12 min, and transferred to holding aCSF at room temperature for at least 1 h before recording. Slices were visualized under IR-DIC optics (Nikon FN1), and pyramidal neurons in Layer V of the prelimbic cortex (PrL) or putative LC neurons on the lateral wall of the fourth ventricle were targeted.

Patch pipettes (3–6 MΩ) were filled with two different intracellular solutions depending on the recording configuration. Voltage-clamp recordings of excitatory postsynaptic current (EPSC), inhibitory postsynaptic current (IPSC), or NMDAR current were acquired with a cesium-based internal solution, pH 7.3 (290 mOsm), containing the following (in mM): 120 Cs-methanesulfonate, 5 CsCl, 10 Na_2_-phosphocreatine, 10 HEPES, 4 Mg-ATP, 0.3 Na-GTP, and 5 QX-314. Current-clamp recordings and assessment of intrinsic membrane properties were acquired with a potassium-based internal solution, pH 7.3 (290 mOsm), composed of the following (in mM): 135K-gluconate, 4 KCl, 2 NaCl, 10 HEPES, 4 EGTA, 4 Mg-ATP, and 0.3 Na-GTP. Series resistance was monitored continuously, and recordings were discarded if resistance changed by >20%. In selected experiments, 0.3% biocytin (Thermo Fisher Scientific, catalog #28022) was added to the internal solution for post hoc morphological identification.

In slices expressing ChR2 in mPFC*→*LC neurons, 473 nm light (1 ms pulses at ∼1 mW/mm^2^) was delivered through the objective. Evoked quantal EPSCs were recorded in the presence of tetrodotoxin (TTX; 1 µM), APV (50 µM), picrotoxin (PTX; 10 µM), and 4-AP (100 µM), with 2 s 470 nm light pulses to evoke quantal release, following protocols adapted from Miska et al. (Miska et al., 2018).

Spontaneous EPSCs (sEPSCs) and IPSCs (sIPSCs) were recorded in standard aCSF while holding the membrane potential at −70 and 0 mV, respectively. Miniature EPSCs (mEPSCs) and IPSCs (mIPSCs) were recorded under the same conditions in the presence of 1 µM TTX to block action potentials (APs). For NMDAR-mediated postsynaptic currents, neurons were voltage clamped at +40 mV in modified recording aCSF (1 mM MgSO_4_) supplemented with CNQX (10 µM) and PTX (10 µM) to block α-amino-3-hydroxy-5-methyl-4-isoxazolepropionic acid (AMPA) and gamma-aminobutyric acid currents, respectively. Recordings were made using an Axoclamp 200B amplifier (Molecular Devices), digitized at 20 kHz, low-pass filtered at 2 kHz, and analyzed offline using Clampfit 11 and custom Python scripts. Recordings were included if series resistance was <17 MΩ with <15% drift; leak current was <−200 pA with <50 pA drift.

### Patch-clamp data analysis

Whole-cell recordings were analyzed using the eFEL Python library and custom scripts (https://github.com/NeuroDataa/PatchClampAnalysis). Voltage traces were low-pass filtered at 1 kHz before analysis.

Active membrane properties—including AP amplitude, width, threshold, and afterhyperpolarization—were measured from a 300 ms depolarizing current step at rheobase. AP threshold was identified based on the time point where the membrane potential's derivative (dV/dt) exceeded 10 mV/ms, capturing the first spike. Passive properties, including membrane time constant (*τ*) and input resistance, were extracted from hyperpolarizing steps using monoexponential curve fitting. Sag amplitude was calculated as the difference between the peak and steady-state voltage during the hyperpolarizing pulse.

Spontaneous EPSCs and IPSCs were detected using a template-matching algorithm in Clampfit, with templates generated by averaging >100 manually confirmed events per condition. A detection threshold of 3.5× root mean square of baseline noise was applied. Event amplitude, frequency, and decay constants were exported and analyzed in Python.

Evoked EPSC decay kinetics were fitted using monoexponential models in Python, and values were averaged per cell. Paired-pulse ratios (PPRs) were calculated as EPSC2/EPSC1. For short interstimulus intervals, the decay of the first EPSC was subtracted from the second to minimize overlap.

### Drugs

NE hydrochloride (item number 35580), yohimbine hydrochloride (item number 19869), prazosin hydrochloride (item number 15023), ICI-118551 hydrochloride (item number 15591), CGP-20712A (item number 40765), NVP-AAM077 (item number 30622), CNQX (item number 14618), and PTX (item number 20771) were purchased from Cayman Chemical. Compounds were dissolved at the specified final concentration in aCSF and added to the bath. Working solutions were prepared from freshly diluted stocks of frozen aliquots. An equivalent concentration of DMSO was added to the vehicle solutions for compounds dissolved in DMSO.

### Multielectrode array (MEA) recordings

Local-field potential recordings were obtained from acute 300 µm coronal mPFC slices using perforated MEAs (pMEAs). Slices were allowed to equilibrate for 15 min prior to baseline acquisition.

NE (10 µM) was bath-applied to induce subthreshold network activity. To assess adrenoceptor involvement, slices were preincubated with the adrenoceptor antagonist yohimbine (α2, 10 µM), prazosin (α1, 100 nM), ICI-118551 (β2, 10 nM), and CGP-20712A (β1, 100 nM) for 10 min. For optogenetic experiments, 473 nm light (5 s pulse at 20 Hz, ∼1 mW/mm^2^) was delivered to the PrL via a digital micromirror device (Polygon-400, Mightex) mounted on a 10× objective (CKX53, Olympus), targeting slices expressing ChR2 in LC→mPFC axons.

Recordings were sampled at 20 kHz and processed using custom Python scripts (https://github.com/NeuroDataa/Grin2a_LC_mPFC_pMEA_Analysis). Signals were bandpass filtered (4–400 Hz) using a second-order Butterworth filter, followed by a 60 Hz notch filter (*Q* = 30), and downsampled to 1 kHz via staged decimation. A 500 Hz low-pass filter was applied for spectral analysis.

Multitaper spectra were computed with a time-bandwidth product of 5 (*K* = 8 tapers). Spectrograms used 4 s windows with a 1 s step. We defined gamma as 30–100 Hz and high-frequency oscillations (HFOs) as 100–200 Hz (Colgin et al., 2009; Huang et al., 2024). Absolute power was obtained by integrating the power spectral density over each band. For each slice, power was averaged across electrodes within a visually defined ROI, and slice means were then averaged to yield genotype- and condition-level means.

For NE pharmacology, 5 min windows before and after drug application were analyzed. For optogenetic stimulation, 1 min pre- and poststimulation windows were used.

### Histology

Tissue preparation, immunostaining, and imaging were performed as described previously (Hosseini et al., 2025). Briefly, mice were deeply anesthetized and transcardially perfused with ice-cold 1× phosphate-buffered saline (PBS), followed by 4% paraformaldehyde. Brains were postfixed overnight at 4°C, cryoprotected in 30% sucrose, and sectioned coronally at 60 μm using a vibrating microtome (PELCO easiSlicer, model number 11000). Free-floating sections were permeabilized and blocked in PBS containing 10% donkey serum and 0.5% Triton X-100 for 1 h at room temperature. Sections were incubated overnight at 4°C with primary antibodies diluted in a blocking buffer. Slices were washed, and secondary antibodies were applied for 2 h at room temperature. The following antibodies were used:

### Antibodies

Primary: Rabbit anti-TH (Millipore, AB152, 1:1,000) and mouse anti-NET (Sigma-Aldrich, AMAb91116, 1:1,000).

Secondary: Alexa Fluor 647 donkey anti-rabbit (Life Technologies, A31573, 1:2,000) and Alexa Fluor 488 donkey anti-mouse (Life Technologies, A21202, 1:2,000).

Sections were mounted on slides and coverslipped with ProLong Gold Antifade Mountant (Molecular Probes #P36930). Imaging was performed using a Zeiss LSM 880 confocal microscope with a 20× objective. Excitation wavelengths were set to 405, 488, 561, and 633 nm, and laser power, gain, and other acquisition settings were kept constant across samples to ensure quantitative comparability.

### Image analysis

Cell counts were performed on 3–4 rostrocaudal sections per animal using ImageJ (https://imagej.nih.gov/ij/). For ChETA expression analysis, eYFP^+^ signals were isolated by splitting channels and converting images to 8 bit grayscale. Regions of interest (ROIs) were manually drawn around the LC region, and cell bodies were segmented using the Otsu thresholding method. Particles ≥20 μm^2^ were analyzed with the “Analyze Particles” tool, extracting metrics including particle count, area, and mean gray value. Mean fluorescence intensity, representing average pixel brightness within segmented ROIs, was a proxy for relative ChETA expression levels. Cell density was quantified as the number of labeled cells per unit area.

To assess both targeting efficiency and specificity within the heterogeneous LC, we quantified two complementary metrics: the proportion of TH^+^ neurons coexpressing the viral fluorophore (eYFP or FusionRed) and, conversely, the proportion of fluorophore-labeled neurons that were TH-positive.

Axonal density was quantified as total axon length (millimeters) per unit tissue area (square millimeters) using the Ridge Detection plugin in ImageJ. Native fluorescence was used to detect mCherry^+^ LC fibers, whereas NET^+^ fibers in the mPFC were visualized by immunohistochemistry prior to density quantification.

### Experimental design and statistical analysis

All experiments compared WT, heterozygous (*Grin2a*^HET^), and homozygous knock-out (*Grin2a*^KO^) mice across behavioral, electrophysiological, and anatomical measures. Sample sizes were based on prior studies using similar reversal learning paradigms (Marquardt et al., 2019). For each experiment, the specific sample size (*n*) and unit of analysis (animals, cells, or slices) are reported in the corresponding figure legends.

Animals were randomly assigned to experimental groups, and experimenters were blinded to genotype during behavioral testing, electrophysiological recordings, and all data analysis. Data were collected from at least two independent cohorts to reduce batch effects and improve reproducibility.

Behavioral performance (sessions to criterion) was analyzed using repeated-measures ANOVA with genotype and condition as factors, with mouse as the repeated measure. The goal arm side was evaluated as a potential confound using two-way ANOVA (genotype × side; data not shown), with Bonferroni-corrected post hoc tests when appropriate.

For electrophysiological experiments involving repeated measurements within slices (e.g., baseline vs stimulation or drug application), data were additionally analyzed using linear mixed-effects models with condition and genotype as fixed effects and slice treated as a random intercept. This approach was used to account for within-slice pairing and hierarchical data structure. Post hoc comparisons were adjusted for multiple testing as indicated in the figure legends. Full model outputs are provided in Extended Data.

All quantitative data are presented as mean ± SEM unless otherwise stated. Statistical tests (parametric or nonparametric) and any post hoc corrections for multiple comparisons are indicated in the figure legends. Box plots depict the median (center line), interquartile range (box), and minimum and maximum values (whiskers). Asterisks denote significance levels: *p* < 0.05; *p* < 0.01; *p* < 0.001.

### Data and code availability

All original analysis code generated for this study has been deposited in publicly accessible GitHub repositories:

https://github.com/NeuroData/PatchClampAnalysis https://github.com/NeuroDataa/MEA_Analysis

This study did not generate new or unique reagents.

## Results

### Reversal learning is impaired in *Grin2a* mutants

**Figure 1.**
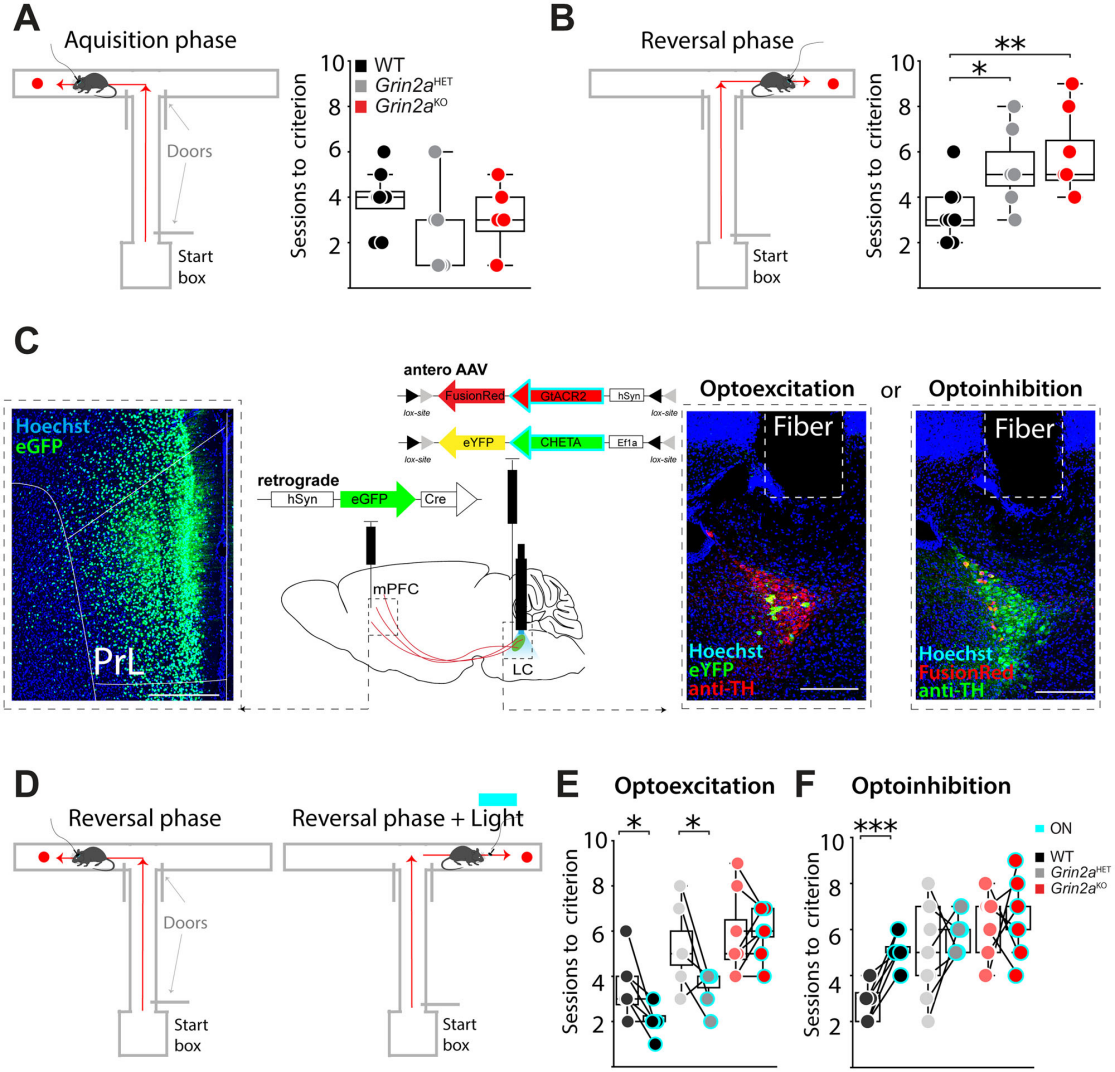
LC→mPFC modulation of reversal learning is disrupted by GluN2A deficiency. ***A***, Acquisition phase schematic*.* Mice were trained to seek a sucrose pellet (red circle) in a custom T-maze with manually controlled doors. All genotypes reached criterion performance (>70% correct choices) during acquisition with no significant differences (Kruskal–Wallis, all *p* > 0.05). ***B***, Reversal learning performance*.* After acquisition, the reward location was switched, requiring animals to adapt to a new contingency. *Grin2a*^HET^ and *Grin2a*^KO^ mice required more sessions to reach criterion than *Grin2a*^WT^ controls (*Grin2a*^WT^, 3.38 ± 0.46; *Grin2a*^HET^, 5.29 ± 0.72; *Grin2a*^KO^, 5.75 ± 0.65; Kruskal–Wallis test *H*_(2)_ = 7.38; *p* = 0.025; Dunn's post hoc *Grin2a*^WT^ *<* *Grin2a*^HET^, *Grin2a*^WT^ *<* *Grin2a*^KO^; *p* *<* 0.05). ***C***, LC→mPFC circuit targeting and optogenetic design. Retrograde Cre virus was injected into mPFC and a Cre-dependent virus into LC (ChETA-eYFP for activation or GtACR2-FusionRed for inhibition). Left, Retrograde Cre expression in mPFC-projecting LC neurons. Middle, Schematic of unilateral optical fiber implantation and stimulation protocols (phasic, 470 nm; 25 Hz bursts for 10 s; sustained, 470 nm continuous for 10 s). Right, TH^+^ LC neurons colabeled with retrograde Cre and either ChETA (green) or GtACR2 (red). Scale bars, mPFC, 500 µm; LC, 100 µm. ***D***, Task design for optogenetic modulation*.* Mice underwent reversal learning before and after unilateral optogenetic LC stimulation. Light delivery began while the mouse was in the start box and ended prior to door opening. ***E***, Phasic LC excitation facilitates reversal learning*.* Phasic LC stimulation improved performance in *Grin2a*^WT^ (2.12 ± 0.23; paired *t*_(7)_ = 2.45; *p* = 0.044) and *Grin2a*^HET^ (3.57 ± 0.31; *t*_(6)_ = 2.38; *p* = 0.048) mice but had no effect in *Grin2a*^KO^ mice (6.00 ± 0.38; *t*_(7)_ = −0.76; *p* = 0.47). ***F***, Sustained LC inhibition impairs reversal learning in WT mice*.* LC inhibition impaired reversal performance in *Grin2a*^WT^ mice (5.12 ± 0.28; paired *t*_(7)_ = −6.15; *p* = 0.0005), but not *Grin2a*^HET^ (5.67 ± 0.24; *t*_(6)_ = −0.81; *p* = 0.44) or *Grin2a*^KO^ (6.44 ± 0.50; *t*_(7)_ = −0.27; *p* = 0.79). Box plots, center line, median; box, 25th–75th percentile; whiskers, min–max. *n* = 7–9 mice per genotype. *p < 0.05; ***p < 0.001. Additional characterization of viral targeting efficiency and specificity is provided in Extended Data Figures 1-1 and 1-2. Optical fiber placements are summarized in Extended Data Figure 1-3.

Reversal learning depends on mPFC function and its modulation by noradrenergic input from the LC (Janitzky et al., 2015). Because GluN2A-containing NMDA receptors are critical for prefrontal network maturation and oscillatory coordination, we first asked whether their deficiency impairs reversal learning in a spatial T-maze task that reliably engages mPFC circuits. This paradigm allowed us to test whether GluN2A deficiency disrupts cognitive flexibility in a behavior tightly linked to LC–mPFC interactions, helping to resolve inconsistent findings from prior studies that used other behavioral assays (Ramos and Arnsten, 2007; Janitzky et al., 2015). During acquisition, all genotypes reached criterion performance (>70% correct across 10 consecutive sessions) at comparable rates, with no significant differences observed (Fig. 1*A*). In contrast, during reversal learning, both *Grin2a*^HET^ and *Grin2a*^KO^ mice required significantly more sessions than *Grin2a*^WT^ controls (WT, 3.38 ± 0.46; HET, 5.29 ± 0.72; KO, 5.75 ± 0.65 sessions; Kruskal–Wallis test; see Fig. 1 legend for statistics; Fig. 1*B*). These results demonstrate that spatial reversal learning is impaired by GluN2A deficiency, consistent with reduced cognitive flexibility, establishing a behavioral phenotype for subsequent circuit-level tests.

10.1523/ENEURO.0050-26.2026.f1.1 Figure 1-1 TH⁺ neuron density and LC projection integrity are preserved in *Grin2a* mutants. **(A)** Representative confocal micrographs of medial prefrontal cortex (mPFC) and locus coeruleus (LC) in *Grin2a*^WT^, *Grin2a*^HET^, and *Grin2a*^KO^ mice. Tissue was immunostained for tyrosine hydroxylase (TH⁺, white), counterstained with Hoechst (blue), and imaged for eYFP (green), which labels LC projection neurons. Yellow arrowheads indicate TH⁺ neurons expressing eYFP. Scale bars: 600 μm (mPFC); 200 μm (LC). **(B) Genotype comparison.** Pie charts showing the relative proportion of eYFP⁺ cells and TH⁺ cells in the LC across genotypes. **(C) Quantification of TH⁺ cell density, percentage of TH⁺ cells co-expressing eYFP, and mean eYFP signal intensity per LC neuron.** Values represent mean ± SEM. n = 2 mice per genotype; three matched sections were analyzed per animal. Related to Figure 1. These data provide additional characterization of TH⁺ neuron density and mPFC-projecting LC neuron labeling across genotypes. Download Figure 1-1, TIF file.

**Figure 2.**
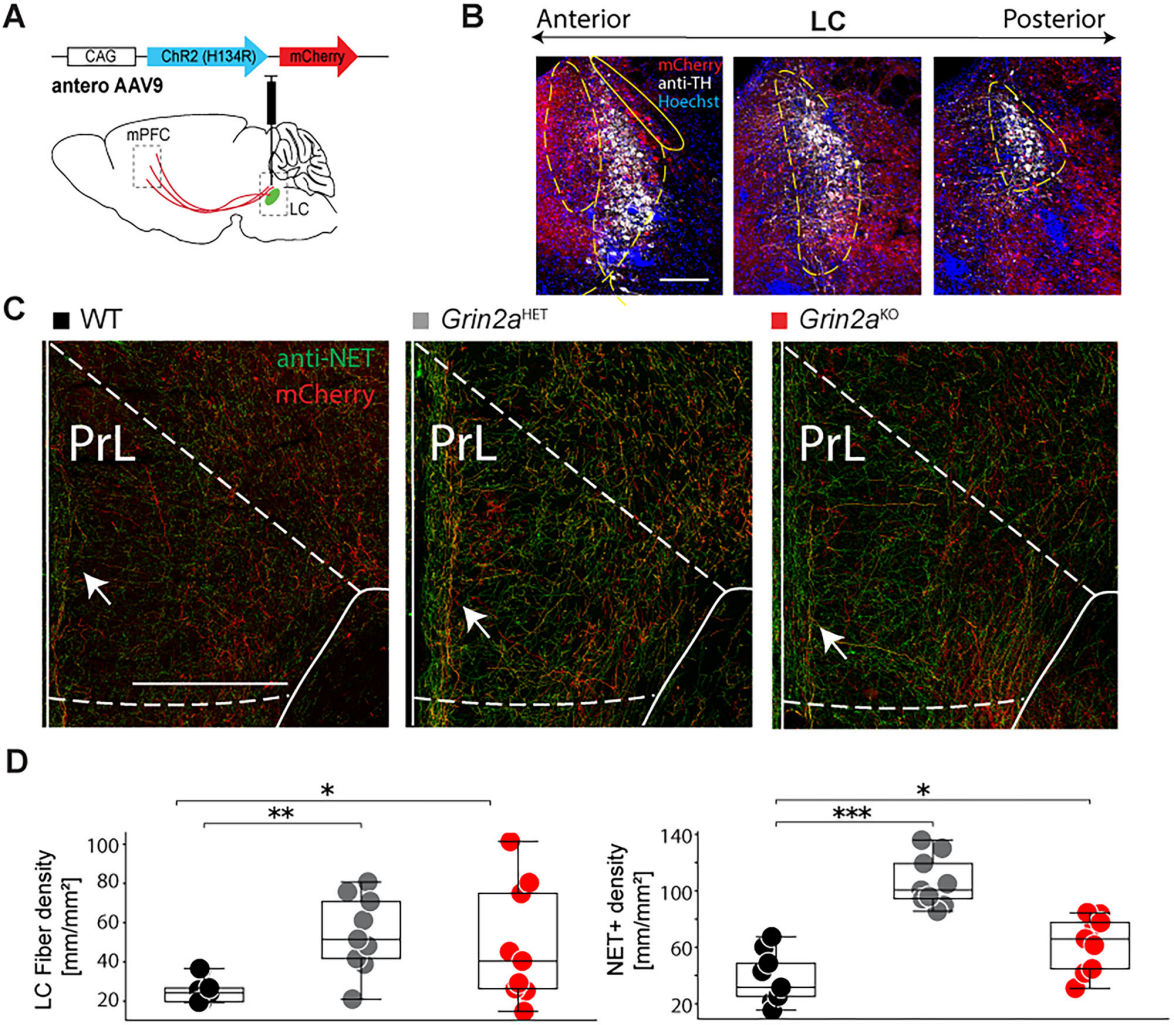
GluN2A deficiency is associated with increased LC–mPFC projection density. ***A***, Viral tracing strategy*.* AAV-mCherry was unilaterally injected into the LC to label noradrenergic neurons projecting to the mPFC. Scale bar, 200 μm. ***B***, LC labeling*.* Representative confocal images showing tyrosine hydroxylase (TH)^+^ neurons (gray) and mCherry-labeled neurons (red) in anterior and posterior LC sections. Scale bar, 200 μm. ***C***, LC→mPFC projection labeling. Confocal micrographs of mCherry + LC axons within the PrL of *Grin2a*^WT^, *Grin2a*^HET^, and *Grin2a*^KO^ mice. Scale bar, 500 μm. ***D***, Quantification of LC fiber density*.* Left, mCherry-labeled LC→mPFC axon density (millimeter/square millimeter): *Grin2a*^WT^ (25.72 ± 2.20), *Grin2a*^HET^ (54.43 ± 6.48), *Grin2a*^KO^ (48.67 ± 9.96); Kruskal–Wallis test, *H*_(2)_ = 9.34; *p* = 0.009; Dunn's post hoc *Grin2a*^WT^ *<* *Grin2a*^HET^ (*p* = 0.009) and *WT* *<* *Grin2a*^KO^ (n.s., *p* = 0.1). Right, NET^+^ fiber density (millimeter/square millimeter): WT (38.13 ± 5.96), *Grin2a*^HET^ (106.31 ± 6.02), *Grin2a*^KO^ (62.96 ± 6.54); Kruskal–Wallis test, *H*_(2)_ = 19.83; *p* = 0.00005; Dunn's post hoc *WT* *<* *Grin2a*^HET^ (*p* = 0.00003) and *WT* *<* *Grin2a*^KO^ (n.s., *p* = 0.35). Box plots, center line, median; box, 25th–75th percentile; whiskers, min–max. Symbols represent per-mouse mean fiber density averaged across visually matched 60 µm coronal sections. Three matched sections were analyzed per mouse. *n* = 3 mice per genotype per assay (mCherry and NET analyses performed in separate cohorts). **p < 0.01; ***p < 0.001. Additional analyses of intrinsic electrophysiological properties, spontaneous synaptic activity, and AMPAR- and NMDAR-mediated transmission in LC neurons are provided in Extended Data Figures 2-1–2-4.

10.1523/ENEURO.0050-26.2026.f1-2 Figure 1-2 **Optogenetic activation and inhibition of mPFC-projecting LC neurons using ChETA and GtACR2.** **(A) ChETA-mediated excitation**. Current-clamp recordings from retrogradely labeled (eYFP⁺) LC neurons expressing ChETA. Continuous 470 nm light stimulation (5 s) induced sustained action potential firing. Bottom: Brief 470 nm light pulses (0.1 ms) delivered at 16, 32, or 64 Hz reliably evoked spiking, with firing rate scaling with stimulation frequency. **(B) GtACR2-mediated inhibition**. Current-clamp recordings from FusionRed⁺ LC neurons expressing GtACR2. A 500 ms pulse of 470 nm light (blue bar) suppressed action potential firing during a 1.5-s depolarizing current step (25 pA), confirming effective optogenetic inhibition of LC neurons. Related to Figure 1. These data validate optogenetic activation and inhibition of mPFC-projecting LC neurons using ChETA and GtACR2. Download Figure 1-2, TIF file.

**Figure 3.**
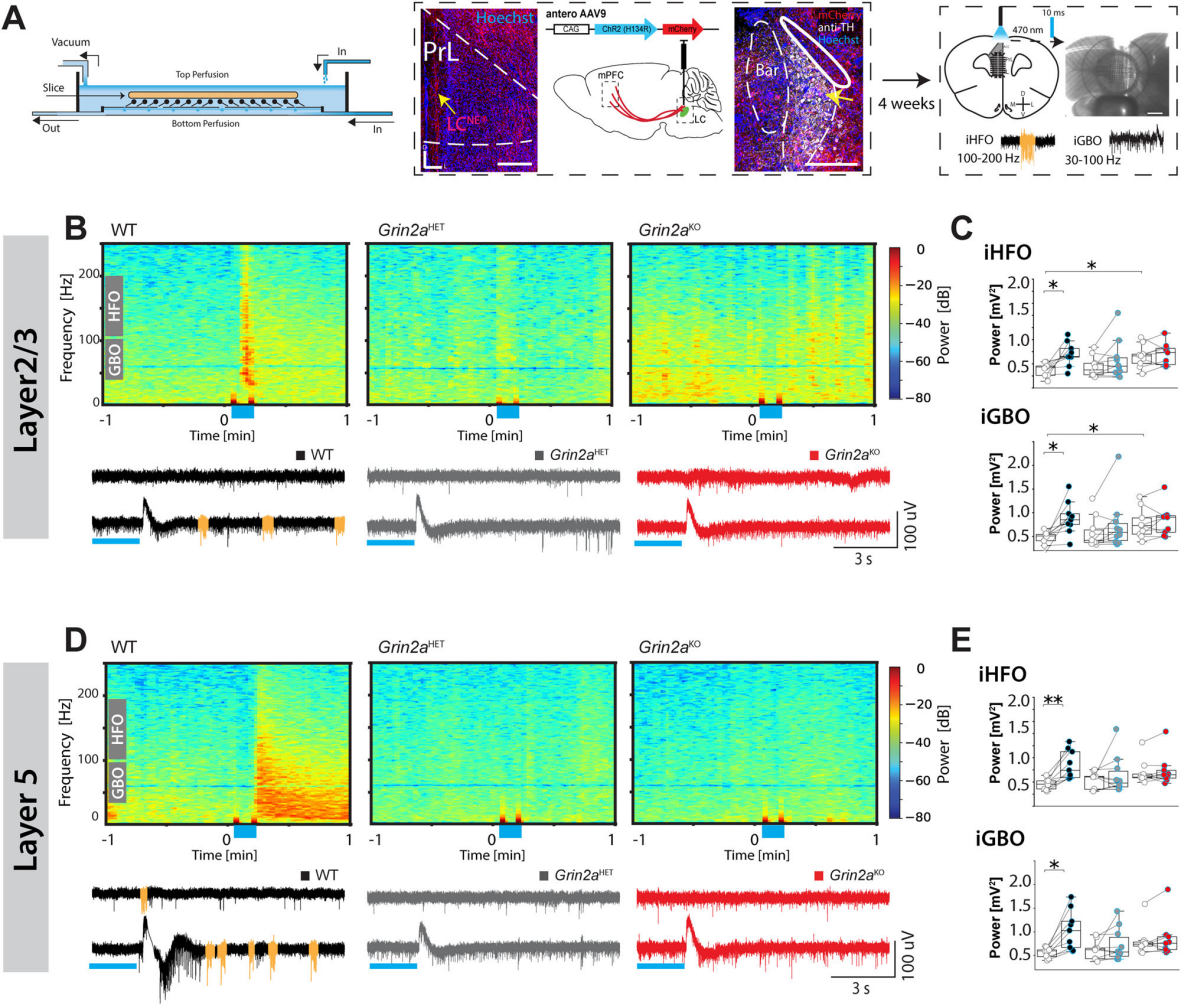
Optogenetic stimulation of LC terminals increases gamma- and high-frequency oscillatory power in WT mPFC slices, with attenuated recruitment in *Grin2a* mutants. ***A***, Experimental setup*.* Left, Schematic of the perfusion-based pMEA system used for ex vivo mPFC recordings. Middle, Confocal image showing ChR2-mCherry^+^ LC terminals in PrL following unilateral injection into the LC. Barrington's nucleus (Bar) indicated for anatomical reference. Scale bar, 100 μm. Right, Schematic showing optogenetic LC→mPFC terminal activation in slices mounted on pMEAs. Representative local-field potential (LFP) traces illustrate light-induced GBO (iGBO, 30–100 Hz) and HFOs (iHFO, 100–200 Hz); HFO bursts highlighted in orange. Scale bar, 1 mm. ***B***, Layer II/III responses. Representative spectrograms and LFP traces from PrL Layer II/III before and after LC terminal stimulation (blue box, stimulation epoch). HFO bursts highlighted in orange. ***C***, Quantification of Layer II/III oscillatory power. iGBO and iHFO power at baseline (open symbols) and after LC terminal stimulation (closed symbols) in *Grin2a*^WT^, *Grin2a*^HET^, and *Grin2a*^KO^ slices. Statistics: linear mixed-effects model (REML) with fixed effects of genotype, condition (baseline vs stimulation), and genotype × condition and slice as a random intercept (paired within-slice measurements). For iHFO (Layer II/III), the overall condition contrast was significant (Tukey, *p*_adj = 0.0236), driven by a significant within-slice increase in *Grin2a*^WT^ (*p*_adj = 0.0042), whereas within-slice changes were not detected in *Grin2a*^HET^ (*p*_adj = 0.3329) or *Grin2a*^KO^ (*p*_adj = 0.6929); the genotype × condition terms were not significant (WT × condition *p* = 0.143; KO × condition *p* = 0.386). For iGBO (Layer II/III), the overall condition contrast was significant (*p*_adj = 0.0295), with a significant within-slice increase in *Grin2a*^WT^ (*p*_adj = 0.0042) but not in *Grin2a*^HET^ (*p*_adj = 0.4228) or *Grin2a*^KO^ (*p*_adj = 0.6897); genotype × condition terms were not significant (WT × condition *p* = 0.069; KO × condition *p* = 0.472). ***D***, Layer V responses. Same as ***B***, but for Layer V recordings. ***E***, Quantification of Layer V oscillatory power. Same as ***C***, but for Layer V. For iHFO (Layer V), the overall condition contrast was significant (*p*_adj = 0.0184), and a genotype × condition interaction was detected for *Grin2a*^WT^ (WT × condition *p* = 0.011), with a significant within-slice increase in *Grin2a*^WT^ (*p*_adj = 0.0013) but not in *Grin2a*^HET^ (*p*_adj = 0.4328) or *Grin2a*^KO^ (*p*_adj = 0.7608). For iGBO (Layer V), the overall condition contrast was significant (*p*_adj = 0.017), and a genotype × condition interaction was detected for *Grin2a*^WT^ (WT × condition *p* = 0.004), with a significant within-slice increase in *Grin2a*^WT^ (*p*_adj = 0.0039) but not in *Grin2a*^HET^ (*p*_adj = 0.3481) or *Grin2a*^KO^ (*p*_adj = 0.7615). Box plots, center line, median; box, 25th–75th percentile; whiskers, min–max. *n* = 28 slices total (8–9 slices from 3 mice per genotype); two observations per slice (baseline and stimulation). Symbols represent the mean value per slice. **p < 0.01. Full mixed-effects statistical analyses are provided in Extended Data Figure 3-1.

10.1523/ENEURO.0050-26.2026.f1-3 Figure 1-3 **Optical fiber placement relative to the locus coeruleus is comparable across genotypes.** Mean distance from optical fiber tips to the locus coeruleus is shown for *Grin2a* WT, HET, and KO mice in experiments using ChETA or GtACR expression in LC neurons. Values are presented as mean ± SEM (µm); n = 7–9 slices from 3 mice per genotype. Statistical analyses were performed using the Kruskal–Wallis test with pairwise comparisons made relative to WT. No significant genotype-dependent differences in optical fiber placement were detected. Related to Figure 1. These data provide verification of optical fiber placement across genotypes. Download Figure 1-3, TIF file.

### LC→mPFC projections bidirectionally modulate reversal learning

Because reversal learning depends on LC-driven modulation of mPFC activity, we next examined whether direct manipulation of LC terminals in mPFC alters performance in a GluN2A-dependent manner. Noradrenergic signaling from the LC shapes cognitive flexibility by modulating mPFC network dynamics (Sara, 2009; McBurney-Lin et al., 2022), and prior work shows that optogenetic or chemogenetic activation of LC neurons enhances reversal and set-shifting performance (Janitzky et al., 2015; Cope et al., 2019; Xiang et al., 2019). To directly test whether LC→mPFC projections contribute to reversal learning, we used a dual-viral approach combining retrograde Cre delivery to the mPFC with Cre-dependent viral constructs in the LC to express ChETA (for activation) or GtACR2 (for inhibition). Circuit specificity was confirmed by comparable TH^+^ LC neuron density and similar proportions/intensity of retrogradely labeled mPFC-projecting LC neurons across genotypes (Fig. 1*C*; Extended Data Fig. 1-1). Expression of ChETA-mCherry in LC neurons and terminal fields within the mPFC was robust and restricted to noradrenergic neurons, confirming successful targeting of the LC→mPFC pathway (Fig. 1*D*). We additionally verified opsin function ex vivo: ChETA-expressing, retrogradely labeled LC neurons showed robust light-evoked spiking across stimulation frequencies, whereas GtACR2 activation reliably suppressed firing during depolarizing drive (Extended Data Fig. 1-2). Optical fiber placement relative to the LC did not differ across genotypes for either the ChETA or GtACR2 cohorts (Extended Data Fig. 1-3).

In *Grin2a*^WT^ mice, phasic activation of LC terminals (470 nm, 25 Hz bursts for 10 s) significantly reduced the number of sessions required to reach criterion (baseline, 3.38 ± 0.46; stimulation, 2.12 ± 0.23; paired *t*_(7)_ = 2.45; *p* = 0.044; see Figure 1 legend for statistics; Fig. 1*E*), whereas sustained photoinhibition (470 nm, 10 s continuous) impaired performance (baseline, 3.38 ± 0.46; inhibition, 5.12 ± 0.28; paired *t*_(7)_ = −6.15; *p* = 0.0005; Fig. 1*F*). Activation also facilitated reversal in *Grin2a*^HET^ mice (baseline, 5.29 ± 0.72; stimulation, 3.57 ± 0.31), whereas no improvement was observed in *Grin2a*^KO^ mice (baseline, 5.75 ± 0.65; stimulation, 6.00 ± 0.38). Because the largest apparent improvements were observed among animals with poorer baseline performance, the magnitude of individual improvement should also be interpreted with regression toward the mean in mind. Likewise, inhibitory effects were abolished in both HET and KO animals (Fig. 1*F*). These findings show that LC→mPFC projections exert bidirectional control over reversal learning in WT mice, with activation effects partially preserved in *Grin2a*^HET^ mice and absent in *Grin2a*^KO^ mice. The reduced behavioral sensitivity to LC→mPFC manipulation in mutants is consistent with altered noradrenergic integration, but this global model does not distinguish among impaired mPFC responsiveness, altered LC function, or broader developmental adaptations outside this projection.

### Enhanced cortical projections and intrinsic properties of LC neurons in *Grin2a* mice

Noradrenergic signaling within medial prefrontal circuits has been implicated in cognitive flexibility and related executive functions (Lapiz and Morilak, 2006). Therefore, we quantified LC innervation and NE transporter (NET) expression in mPFC and assessed whether GluN2A deficiency alters LC cellular and synaptic properties (Lapiz and Morilak, 2006; Seu et al., 2009).

To determine whether GluN2A deficiency alters LC→mPFC connectivity or intrinsic LC neuron properties, we quantified axonal projection density and recorded from identified TH^+^ LC neurons. Immunohistochemistry revealed increased LC innervation within the prelimbic cortex (PrL), with mCherry-labeled LC→mPFC fiber density elevated in *Grin2a*^HET^ mice relative to WT and NET^+^ fiber density likewise increased (Fig. 2*D*). *Grin2a*^HET^ mice also exhibited a greater proportion of eYFP-labeled LC neurons that co-expressed TH compared with WT and KO mice (Extended Data Fig. 1-1). Together, these findings are consistent with compensatory remodeling of the noradrenergic LC→mPFC circuit in the setting of partial GluN2A deficiency.

10.1523/ENEURO.0050-26.2026.f2-1 Figure 2-1 Intrinsic electrophysiological properties of LC neurons are largely preserved in *Grin2a* mutants. **(A) Representative recordings.** Current-clamp traces from LC neurons in *Grin2a*^WT^, *Grin2a*^HET^, and *Grin2a*^KO^ mice showing repetitive firing evoked by 300 ms depolarizing current injections. **(B) Input–output relationships.** Firing rate plotted as a function of current injection (−125 to +475 pA, 25 pA step size), applied uniformly across all recorded neurons. **(C) Passive membrane properties.** Quantification of resting membrane potential (V_m_), input resistance (R_in), and membrane capacitance. **(D) Action potential waveforms.** Representative AP traces from LC neurons across genotypes. **(E) Action potential kinetics.** Quantification of AP amplitude, threshold, rise time, decay time, and afterhyperpolarization (AHP). Threshold values were defined as the potential where dV/dt exceeded 10 mV/ms above baseline. Box plots: center line = median; box = 25th–75th percentile; whiskers = min–max. Symbols represent 32–40 cells per genotype derived from 6 mice per group; statistical analyses were performed on per-mouse averages (n = 6/genotype) using Kruskal–Wallis tests. *p < 0.05, **p < 0.01, ***p < 0.001, ****p < 0.0001. Related to Figure 2. These data provide additional characterization of intrinsic electrophysiological properties of LC neurons across genotypes. Download Figure 2-1, TIF file.

10.1523/ENEURO.0050-26.2026.f2-2 Figure 2-2 Spontaneous excitatory, inhibitory, and NMDA receptor-mediated currents in LC neurons of *Grin2a* mutants. **(A) Spontaneous synaptic activity.** Whole-cell patch-clamp recordings from LC neurons in *Grin2a*^WT^, *Grin2a*^HET^, and *Grin2a*^KO^ mice. Spontaneous excitatory postsynaptic currents (sEPSCs) were recorded at −70 mV, and spontaneous inhibitory postsynaptic currents (sIPSCs) were recorded at 0 mV. **(B) Event frequency.** Box plots comparing sEPSC and sIPSC frequencies across genotypes. **(C, D) Event kinetics**. Example traces and quantitative analysis of sEPSCs (**C**) and sIPSCs (**D**). Decay time constants were derived from individually detected events using template-matching algorithms in Clampfit. **(E) Spontaneous NMDA receptor–mediated currents.** sNMDA currents were recorded at +40 mV under pharmacological isolation in modified aCSF containing 1 mM Mg²⁺, CNQX (10 µM), and picrotoxin (10 µM). **(F) sNMDA amplitude and decay.** Representative sNMDA traces (left) and box plots (right) showing amplitude and decay across genotypes. Box plots: center line = median; box = 25th–75th percentile; whiskers = min–max. Symbols represent 11–14 cells, derived from 3 mice per genotype. Statistical analyses were performed on per-mouse averages (n = 3/genotype) using the Kruskal–Wallis test; ** *p* < 0.01, *** *p* < 0.001. Related to Figure 2. These data characterize spontaneous excitatory, inhibitory, and NMDA receptor-mediated synaptic transmission in LC neurons across genotypes. Download Figure 2-2, TIF file.

10.1523/ENEURO.0050-26.2026.f2-3 Figure 2-3 **AMPAR transmission at mPFC→LC synapse.** **(A) Viral labeling and anatomy.** Left: Confocal image showing AAV2-hSyn-ChR2(H134R)-eYFP injection into the mPFC. Middle: Schematic of viral injection strategy and projections from mPFC to noradrenergic neurons in the locus coeruleus (LC). Right: Confocal image of a biocytin-filled LC-NE neuron targeted for whole-cell recording. Scale bars = 500 μm (mPFC); 100 μm (LC); inset = 20 μm. (**B**) **Recording configuration.** Diagram of whole-cell patch-clamp recording from ChR2-expressing mPFC→LC synapses. (**C**) **AMPA-mediated sEPSCs.** Representative traces of excitatory postsynaptic currents (eEPSCs) recorded at −70 mV in standard aCSF across genotypes. (**D**) **eEPSC quantification.** Box plots summarize eEPSC amplitude, response latency, and decay time constant (τ). *Grin2a* mutants exhibited significantly shorter response latencies, and *Grin2a*^HET^ slices showed faster decay τ values compared with *Grin2a*^WT^. (**E, F**) **Paired-pulse ratios.** AMPA-mediated paired-pulse ratios (PPR = eEPSC_2_/eEPSC_1_) evoked at interstimulus intervals (ISIs) of 25, 50, and 100 ms, plotted as a function of ISI. (**G**) **Miniature EPSCs.** Representative traces of quantal AMPA-mediated mEPSCs recorded at −70 mV following stimulation of adjacent ChR2-expressing mPFC→LC axon terminals (470 nm, 2 s). Recordings were performed in the presence of TTX (1 µM), APV (50 µM), picrotoxin (10 µM), and 4-AP (100 µM). (**H, I**) **Quantal analysis.** Box plots show quantal event frequency (Hz) and amplitude (pA), and histograms display frequency–amplitude distributions across genotypes. Box plots: center line = median; box = 25th–75th percentile; whiskers = min–max. Symbols represent 10–15 cells, derived from 3 mice per genotype. Statistical analyses were performed on per-mouse averages (n = 3/genotype) using the Kruskal–Wallis test; ** *p* < 0.01, *** *p* < 0.001. Related to Figure 2. These data compare AMPAR function at mPFC→LC synapses across genotypes. Download Figure 2-3, TIF file.

**Figure 4.**
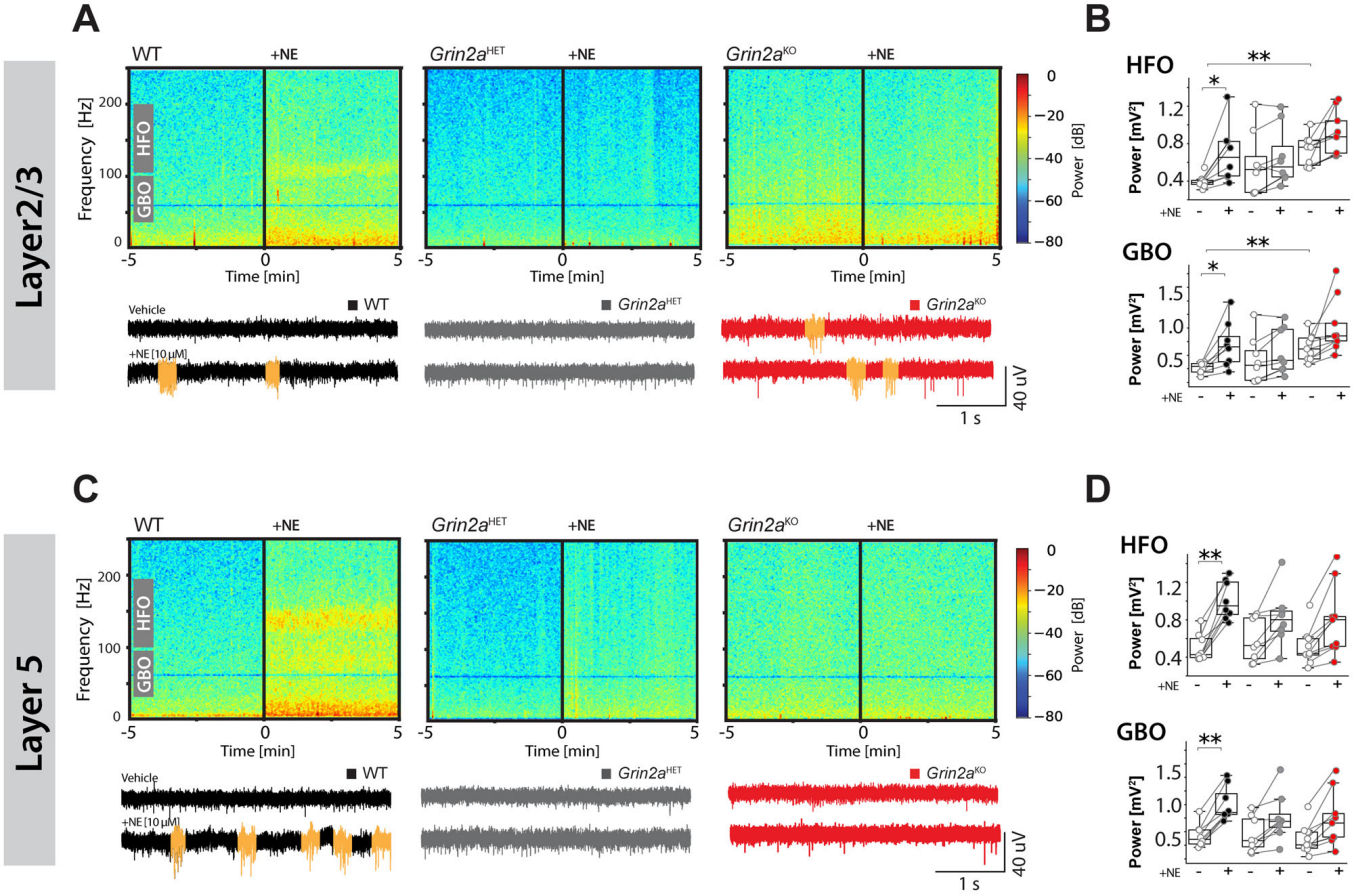
Exogenous NE enhances gamma- and high-frequency oscillations in mPFC slices, with attenuated recruitment in *Grin2a* mutants. ***A***, ***C***, Representative spectrograms and LFP recordings. Bath application of NE (10 µM, 5 min) to acute mPFC slices from *Grin2a*^WT^, *Grin2a*^HET^, and *Grin2a*^KO^ mice. Recordings were obtained from Layer II/III (***A***) and Layer V (***C***) of the PrL. NE elicited prominent bursts of HFOs (100–200 Hz, highlighted in orange) in *Grin2a*^WT^ slices, with reduced recruitment in *Grin2a* mutants. ***B, D***, Quantification of oscillatory power*.* Box plots show GBO power (30–100 Hz) and HFO power (100–200 Hz) in vehicle (open symbols) and NE application (closed symbols) in Layer II/III (***B***) and Layer V (***D***) of PrL for *Grin2a*^WT^, *Grin2a*^HET^, and *Grin2a*^KO^ slices. Statistics: linear mixed-effects model (REML) with fixed effects of genotype, condition (vehicle vs NE), and genotype × condition, and slice as a random intercept (paired within-slice measurements). Post hoc (Tukey) contrasts were used for within-genotype NE versus baseline/vehicle comparisons. In *Grin2a*^WT^ slices, NE increased oscillatory power across measures (Layer II/III, HFO *p*_adj = 0.0167; GBO *p*_adj = 0.0148; Layer V, HFO *p*_adj = 0.0001; GBO *p*_adj = 0.0006). In *Grin2a*^HET^ slices, corresponding within-slice NE effects were not detected (Layer II/III, HFO *p*_adj = 0.6134; GBO *p*_adj = 0.4407; Layer V, HFO *p*_adj = 0.0911; GBO *p*_adj = 0.1232). In *Grin2a*^KO^ slices, within-slice NE effects were attenuated, with a modest increase detected for Layer II/III HFO (*p*_adj = 0.0464) but not for the remaining outcomes (Layer II/III, GBO *p*_adj = 0.0630; Layer V, HFO *p*_adj = 0.0642; GBO *p*_adj = 0.0719). Box plots, center line, median; box, 25th–75th percentile; whiskers, min–max. *n* = 28 slices total (8–9 slices from 3 mice per genotype); two observations per slice (baseline and stimulation). Symbols represent the mean value per slice. *p* < 0.05; **p < 0.01. Full mixed-effects statistical analyses are provided in Extended Data Figure 4-1.

10.1523/ENEURO.0050-26.2026.f2-4 Figure 2-4 **NMDAR transmission at mPFC→LC synapses.** **(A) Spontaneous synaptic activity.** Whole-cell patch-clamp recordings from LC neurons in *Grin2a*^WT^, *Grin2a*^HET^, and *Grin2a*^KO^ mice. Spontaneous excitatory postsynaptic currents (sEPSCs) were recorded at −70 mV, and spontaneous inhibitory postsynaptic currents (sIPSCs) were recorded at 0 mV. **(B) Event frequency.** Box plots comparing sEPSC and sIPSC frequencies across genotypes. **(C, D) Event kinetics**. Example traces and quantitative analysis of sEPSCs (**C**) and sIPSCs (**D**). Decay time constants were derived from individually detected events using template- matching algorithms in Clampfit. Related to Figure 2. These data compare NMDAR function at mPFC→LC synapses across genotypes. Download Figure 2-4, TIF file.

We next examined whether GluN2A deficiency alters intrinsic LC excitability in a manner that could influence noradrenergic output. Across genotypes, LC neurons exhibited broadly preserved firing behavior and input–output relationships, with resting membrane potential, input resistance, and capacitance largely comparable (Extended Data Fig. 2-1). These findings indicate that GluN2A deficiency does not substantially alter baseline intrinsic excitability of LC neurons, suggesting that increased LC→mPFC projection density reflects circuit-level reorganization rather than major changes in LC firing capacity.

We next assessed whether synaptic inputs to LC neurons were altered. Spontaneous excitatory transmission was similar across genotypes, whereas inhibitory event frequency was elevated in *Grin2a*^HET^ neurons, and pharmacologically isolated spontaneous NMDA currents showed prolonged decay with reduced amplitudes in KO neurons (Extended Data Fig. 2-2).

Because LC output is shaped not only by local synaptic inputs but also by reciprocal excitatory feedback from the mPFC, we next tested evoked transmission at identified mPFC→LC synapses using optogenetic stimulation of ChR2-expressing cortical afferents (Extended Data Fig. 2-3, including viral injection/recording schematic). AMPAR-mediated eEPSC amplitudes were comparable across genotypes, but response latencies were shorter in mutants and decay kinetics were modestly accelerated in *Grin2a*^HET^ slices, while PPRs and quantal measures were unchanged, indicating preserved overall excitatory drive with altered temporal features (Extended Data Fig. 2-3).

We then assessed NMDA receptor-mediated transmission at the same pathway. NMDA-mediated eEPSCs recorded under pharmacological isolation revealed reduced amplitudes and prolonged decay kinetics in *Grin2a*^KO^ slices compared with WT (Extended Data Fig. 2-4).

Together, these findings are consistent with bidirectional remodeling within the LC–mPFC circuit, including increased LC innervation of mPFC alongside altered timing and NMDA-dependent integration of cortical feedback to LC. We therefore asked whether noradrenergic drive can still effectively engage prefrontal circuit dynamics by optogenetically stimulating LC terminals in mPFC and measuring induced oscillatory activity.

### LC–NE-induced oscillations in the mPFC are disrupted in *Grin2a* mutants

Gamma-band oscillations (GBO) in the mPFC support cognitive flexibility by synchronizing activity across cortical layers (Breton-Provencher and Sur, 2019; Fan et al., 2022). NE released from LC terminals can drive both GBO (30–100 Hz) and HFO (100–200 Hz) in cortical networks (Neves et al., 2018; Özçete et al., 2024). To test whether LC→mPFC inputs engage these rhythms in a GluN2A-dependent manner, we performed MEA recordings from acute mPFC slices with ChR2 selectively expressed in LC terminals (Fig. 3*A*).

10.1523/ENEURO.0050-26.2026.f3-1 Figure 3-1 **Mixed-effects analysis of optogenetically induced oscillatory power following LC terminal stimulation in mPFC slices.** Results of linear mixed-effects models assessing changes in oscillatory power evoked by optogenetic stimulation of LC terminals in mPFC slices from WT, *Grin2a*^HET^, and *Grin2a*^KO^ mice. Slice identity was included as a random intercept, with fixed effects of genotype, stimulation condition (baseline vs stimulation), and genotype × condition interaction. Reported Δ values reflect Tukey-adjusted within-genotype contrasts (stimulation − baseline) with 95% confidence intervals and multiplicity-adjusted p values. Δ values are reported in mV². Related to Figure 3. These data provide the full mixed-effects statistical analyses supporting the effects of LC terminal stimulation on oscillatory power in mPFC slices. Download Figure 3-1, DOCX file.

Optogenetic stimulation of LC terminals (470 nm, 25 Hz bursts for 10 s) increased GBO and HFO power in WT mPFC slices, whereas corresponding within-slice increases were attenuated and not statistically detected in *Grin2a*^HET^ or *Grin2a*^KO^ slices (Fig. 3*B*–*E*; Extended Data Fig. 3-1). Mixed-effects analysis accounting for paired within-slice measurements (slice as a random effect; Tukey post hoc) indicated genotype-dependent modulation, with the most robust stimulation-linked effects observed in Layer V (see Fig. 3 legend for statistics).

These data indicate that LC terminal stimulation engages mPFC oscillatory activity most reliably in WT slices, with attenuated recruitment in *Grin2a* mutants.

### Exogenous NE induces oscillations in WT mPFC but is altered in *Grin2a* mutants

To bypass LC input and directly test adrenergic signaling, we applied NE to acute mPFC slices and used this preparation to identify receptor subtypes underlying NE-dependent network activation.

NE increased gamma-band and high-frequency power most reliably in WT slices. Across outcomes, within-slice NE effects were not detected in *Grin2a*^HET^ and were attenuated in *Grin2a*^KO^ (Fig. 4*D*; Extended Data Fig. 4-1). Mixed-effects modeling of paired within-slice data supported genotype-dependent NE modulation, with genotype × condition effects prominent in Layer V and evident for HFO in Layer II/III.

10.1523/ENEURO.0050-26.2026.f4-1 Figure 4-1 **Mixed-effects analysis of norepinephrine-evoked oscillatory power in mPFC slices.** Results of linear mixed-effects models assessing changes in oscillatory power evoked by bath application of norepinephrine (NE) in mPFC slices from WT, *Grin2a*^HET^, and *Grin2a*^KO^ mice. Slice identity was included as a random intercept, with fixed effects of genotype, condition (baseline vs NE), and genotype × condition interaction. Reported Δ values reflect Tukey-adjusted within-genotype contrasts (NE − baseline) with 95% confidence intervals and multiplicity-adjusted p values. Δ values are reported in mV² to match descriptive units. Related to Figure 4. These data provide the full mixed-effects statistical analyses supporting the effects of norepinephrine application on oscillatory power in mPFC slices. Download Figure 4-1, DOCX file.

**Figure 5.**
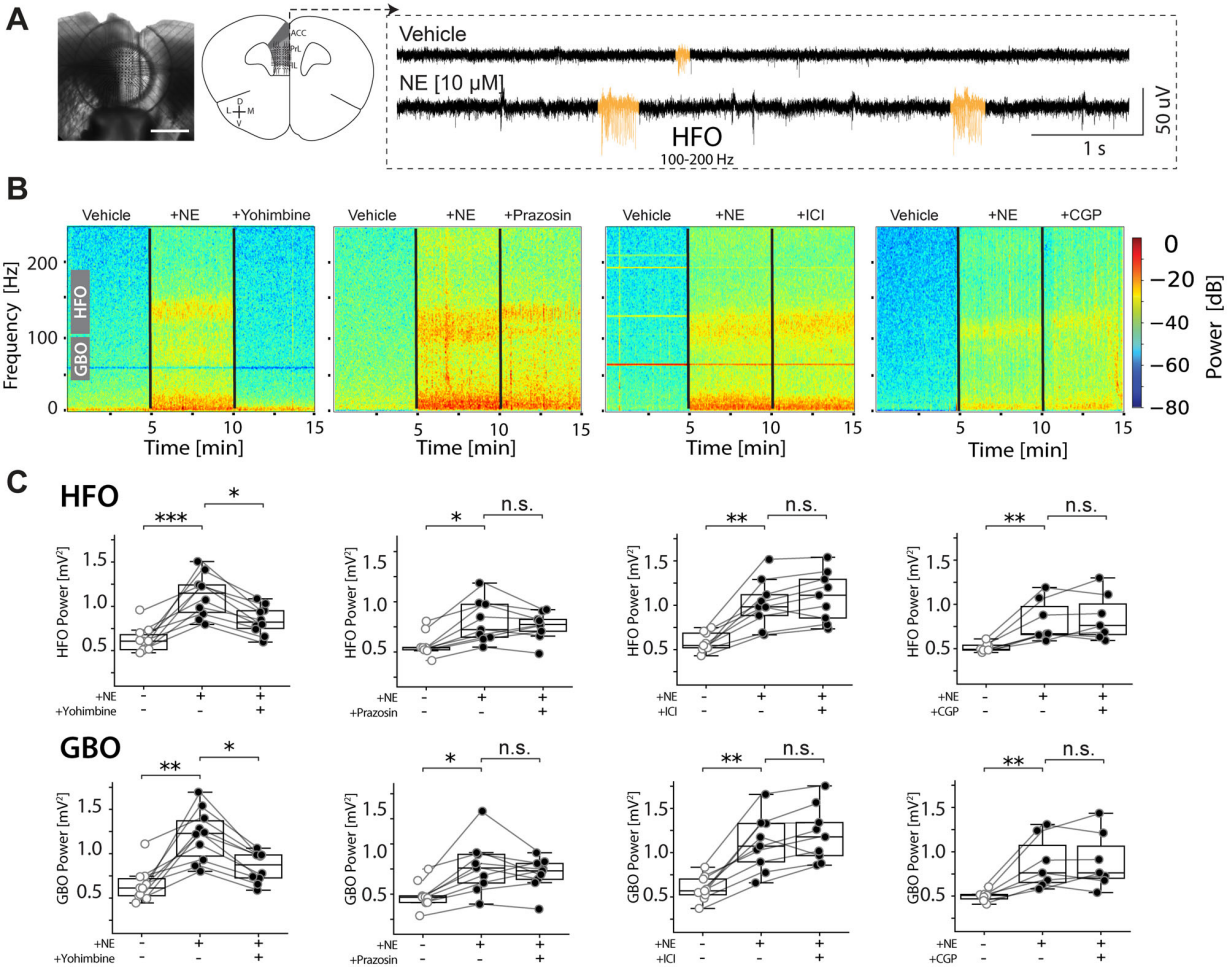
NE-induced GBO and HFOs in mPFC Layer V require α_2_-adrenergic receptor signaling. ***A***, Recording configuration. Left, Bright-field image of an ex vivo mPFC slice mounted on a pMEA chip (scale bar, 1 mm). Middle, Schematic indicating electrode position in Layer V of the PrL. Right, Representative LFP traces recorded during vehicle and during NE (10 µM) application. Bursts of HFO (100–200 Hz) highlighted in orange. ***B***, Adrenergic receptor subtype pharmacology. Representative spectrograms from Layer V PrL recordings showing oscillatory activity after NE application and subsequent coapplication of selective adrenergic receptor antagonists: yohimbine (α_2_, 10 µM), prazosin (α_1_, 100 nM), ICI-118551 (β_2_, 10 nM), or CGP-20712A (β_1_, 100 nM). ***C***, Quantification of NE-induced oscillatory power. Box plots show gamma-band (30–100 Hz) and HFO (100–200 Hz) power per slice during defined post-treatment recording windows. NE-induced oscillations were significantly attenuated by α_2_ blockade with yohimbine, but not by α_1_-, β_1_-, or β_2_-selective antagonists. (HFO, Kruskal–Wallis test, *H*_(4)_ = 16.94; *p* = 0.0002; GBO, Kruskal–Wallis test, *H*_(4)_ = 15.95; *p* = 0.00034; Dunn's post hoc comparisons with Holm–Bonferroni correction, NE vs NE + yohimbine; *p* *<* *0.05*). Box plots, center line, median; box, 25th–75th percentile; whiskers, min–max. Symbols represent the mean value per slice (quantified over the defined post-treatment window). *n* = 8–9 slices from 3 WT mice; each slice received NE and one antagonist condition. *p < 0.05; **p < 0.01; ***p < 0.001.

To identify the receptor mechanisms mediating NE-induced oscillations, we coapplied selective antagonists of α_1_-, α_2_-, β_1_-, or β_2_-adrenergic receptors in WT slices (Fig. 5*B,C*; Kruskal–Wallis test with Dunn's post hoc; see Fig. 5 legend for statistics). Of these, only α_2_-adrenergic receptor blockade with yohimbine significantly reduced NE-induced oscillatory power (see Fig. 5 legend for statistics) indicating that α2 receptors are required for this response.

Together, these findings indicate that NE engages cortical oscillations through α2-adrenergic receptor signaling in WT mPFC and that NE-dependent oscillatory recruitment is reduced in *Grin2a* mutants.

### Acute GluN2A blockade fails to replicate oscillatory deficits in *Grin2a* mutants

**Figure 6.**
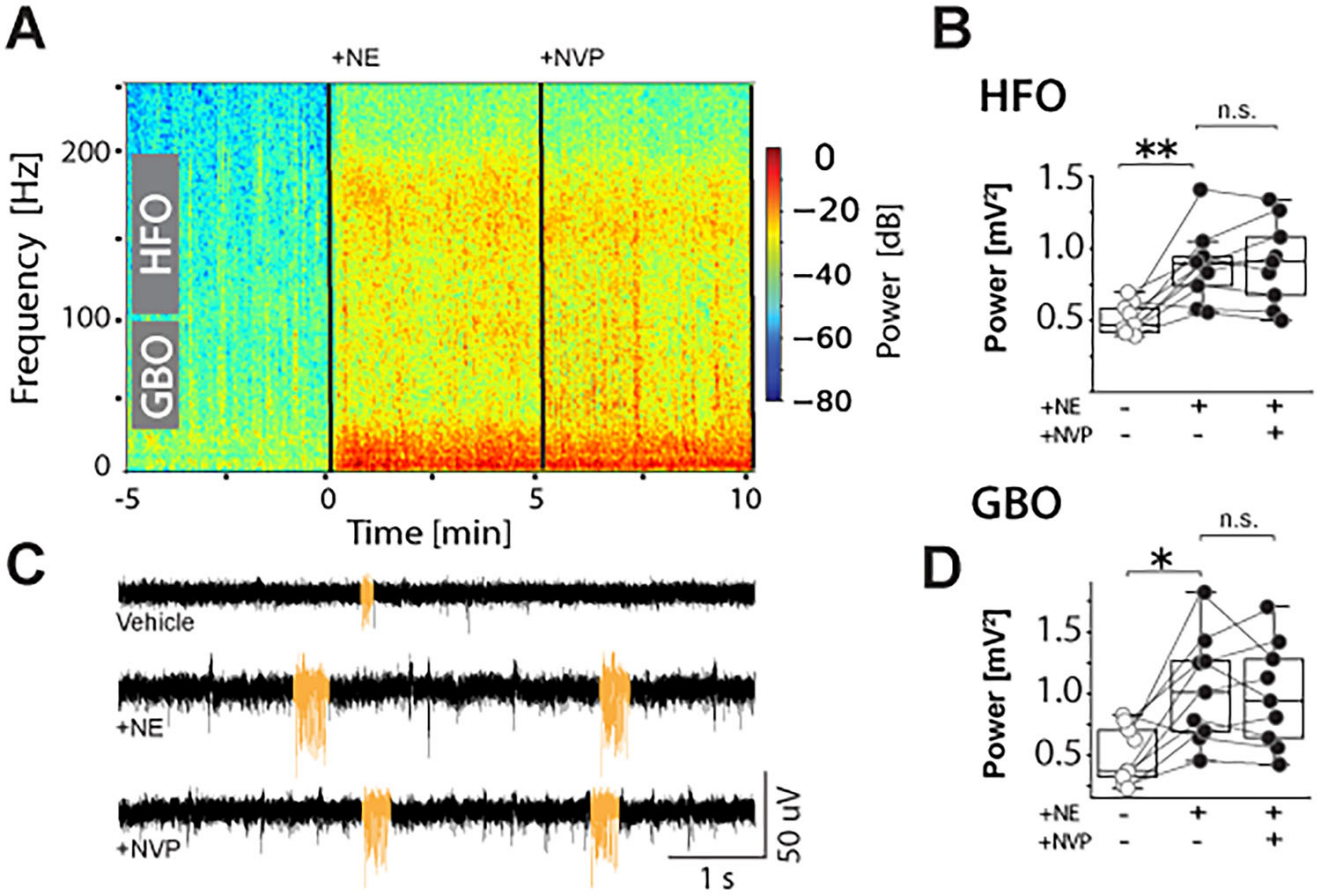
Acute GluN2A blockade fails to disrupt NE-induced gamma- or high-frequency oscillations in mPFC Layer V. ***A***, Representative spectrogram and LFP trace. Oscillatory activity recorded from a Layer V electrode in WT PrL slice during bath application of vehicle (aCSF), NE (10 µM), and NE + NVP-AAM077 (0.5 µM). Oscillatory power was quantified over a 5 min window following each condition. ***B***, Representative LFP traces. Example recordings from a Layer V electrode under NE alone and NE + NVP conditions. Bursts of HFOs (100–200 Hz) are highlighted in orange. ***C***, Quantification of HFO power. HFO (100–200 Hz) power across conditions. Mean ± SEM, HFO (millivolts squared)—Vehicle (0.409 ± 0.029), NE (0.703 ± 0.069), NE + NVP (0.721 ± 0.078). NE significantly increased oscillatory power relative to vehicle, and coapplication of NVP-AAM077 did not reduce NE-evoked HFO activity (Friedman test, *χ*^2^_(2)_ = 8.22; *p* = 0.016; post hoc Wilcoxon signed-rank tests with Holm–Bonferroni correction: Vehicle vs NE, *p* = 0.023; NE vs NE + NVP, *p* = 0.910). ***D***, Quantification of GBO power. GBO (30–100 Hz) power across conditions. Mean ± SEM (millivolts squared)—Vehicle (0.150 ± 0.015), NE (0.258 ± 0.029), NE + NVP (0.248 ± 0.028). NE significantly increased GBO power relative to vehicle, and coapplication of NVP-AAM077 did not reduce NE-evoked GBO activity (Friedman test, *χ*^2^_(2)_ = 8.22; *p* = 0.016; post hoc Wilcoxon signed-rank tests with Holm–Bonferroni correction: Vehicle vs NE, *p* = 0.021; NE vs NE + NVP, *p* = 0.820). Vehicle note. For experiments using DMSO-solubilized antagonists (CNQX, PTX, or CGP-20712A), all bath solutions (including NE-only) contained equivalent DMSO concentrations. NVP-AAM077 was prepared in water and did not introduce DMSO. Box plots, center line, median; box, 25th–75th percentile; whiskers, min–max. Symbols represent the mean value per slice (quantified over the defined post-treatment window). *n* = 9 slices from 3 WT mice. *p < 0.05.

To test whether acute inhibition of GluN2A-containing NMDA receptors can explain the oscillatory deficits observed in *Grin2a* mutants, we applied the GluN2A-selective antagonist NVP-AAM077 during NE application in WT mPFC slices. Sequential bath application of vehicle, NE, and NE + NVP revealed a robust increase in both gamma-band (GBO, 30–100 Hz) and high-frequency (HFO, 100–200 Hz) power following NE relative to vehicle, and oscillatory power was not reduced when NVP was coapplied (Fig. 6*A*–*D*; Friedman test with Wilcoxon post hoc comparisons; see Fig. 6 legend for statistics). These results indicate that acute GluN2A inhibition is insufficient to disrupt NE-driven oscillations, consistent with the idea that the deficits in *Grin2a* mutants reflect long-term circuit or developmental adaptations rather than an acute loss of GluN2A function.

## Discussion

### Summary of key findings

Our findings show that global GluN2A deficiency alters noradrenergic modulation of prefrontal circuits and is associated with impaired cognitive flexibility. Using behavioral, optogenetic, pharmacological, and electrophysiological approaches, we show that LC→mPFC projections bidirectionally regulate reversal learning in WT mice, whereas sensitivity to this neuromodulatory control is reduced in Grin2a mutants.

This behavioral insensitivity occurs alongside impaired NE-evoked prefrontal network synchronization and structural changes in the noradrenergic projection, including increased NET expression and greater LC axonal density in PrL. Together, these findings suggest that global GluN2A deficiency perturbs a reciprocal LC–mPFC loop that normally couples ascending neuromodulatory drive to local prefrontal computations during flexible behavior.

### LC–mPFC neuromodulatory control of cognitive flexibility

Reversal learning depends on coordinating long-range neuromodulatory input with local prefrontal computations (Cools and Arnsten, 2022). In WT mice, optogenetic activation of LC→mPFC projections enhanced reversal performance, whereas inhibition impaired it. In *Grin2a*^KO^ mice, neither LC activation nor inhibition altered reversal learning at the group level. *Grin2a*^KO^ animals nevertheless showed heterogeneous responses to LC manipulation, consistent with residual but unreliable LC–mPFC influence rather than a silent pathway.

*Grin2a*^HET^ mice showed partial behavioral responsiveness to LC activation, yet NE application failed to induce cortical oscillations ex vivo. This dissociation suggests that while LC output may still influence behavior, the prefrontal microcircuits that normally translate NE into coordinated network synchronization are compromised. The lack of behavioral effects during LC inhibition in both *Grin2a*^HET^ and *Grin2a*^KO^ mice may reflect reduced baseline noradrenergic influence and/or diminished sensitivity to transient suppression of LC output, potentially exacerbated by elevated LC resting membrane potential and increased tonic activity. Reduced NE sensitivity within mPFC would further limit LC efficacy, jointly contributing to blunted behavioral responsiveness.

The structural findings further suggest that impaired function is accompanied by noradrenergic remodeling rather than simple loss of LC input. Increased LC axonal density and NET expression in PrL may reflect compensatory reorganization in response to reduced functional integration of LC-derived NE signals within mPFC networks. At the same time, greater innervation and transporter expression do not establish the direction of extracellular NE change during behavior; these adaptations could alter release, clearance, or the temporal precision of neuromodulatory signaling in ways that are not resolved by the present experiments.

Although our projection-specific viral strategy restricted opsin expression to LC→mPFC neurons and achieved comparable targeting across genotypes, GluN2A is expressed throughout the brain. The reduced behavioral sensitivity to LC→mPFC manipulation in Grin2a mutants may therefore reflect impaired neuromodulatory integration within prefrontal networks, altered LC physiology or feedback, developmental changes in other circuits that support reversal learning, or a combination of these mechanisms. Consistent with prior work, *Grin2a*^KO^ mice show deficits in cognitive flexibility despite intact performance on simpler instrumental learning tasks (Brigman et al., 2008). Our findings extend this observation by identifying LC→mPFC signaling as one neuromodulatory pathway altered in the setting of global GluN2A deficiency.

At the circuit level, *Grin2a*^KO^ mutants fail to translate noradrenergic input into gamma-band activity whether NE is released synaptically by LC terminal stimulation or applied exogenously in the bath. This phenotype could reflect disruption of α2R-dependent microcircuits and/or downstream cortical integration; however, our study does not directly test whether α2R expression or signaling is altered in mutants. Independent transcriptomic profiling of the same model argues against a simple loss of receptor transcripts, as *Adra2a* expression is broadly preserved across major PFC cell classes, while transcripts governing GPCR signal termination and effector coupling are altered (Farsi et al., 2023). These alterations could blunt the network impact of NE even when *Adra2a* transcripts are preserved. Direct tests of α2R coupling and downstream effector engagement will be needed to localize the locus of failure.

Complementing this impairment of ascending neuromodulatory drive, optogenetic stimulation of mPFC projections revealed altered excitatory synaptic responses in LC neurons, indicating weakened top–down cortical control of LC activity. LC neurons from *Grin2a* mutants also exhibited a depolarized resting membrane potential and prolonged NMDA current kinetics, consistent with altered intrinsic excitability and reduced temporal precision of synaptic integration. Together, these convergent deficits argue against a purely feedforward dysfunction model and support bidirectional instability in which GluN2A deficiency disrupts both ascending neuromodulatory output and descending cortical feedback, degrading the ability of the LC–mPFC loop to support flexible behavioral control. In this light, GluN2A emerges as a convergence point between developmental circuit refinement and adult neuromodulatory function.

### Developmental disruption of NE circuitry

Our findings suggest that GluN2A contributes to the developmental establishment of NE-sensitive microcircuits required for gamma oscillations and HFOs. In *Grin2a* mutants, NE failed to reliably induce oscillatory activity, whereas α2-adrenergic receptor blockade in WT slices abolished NE-induced oscillations, confirming α2R dependence. However, acute pharmacological inhibition of GluN2A with NVP-AAM077 did not detectably reduce oscillation induction during NE application in WT slices . Together, these results support the view that GluN2A is required for the assembly or maturation of NE-sensitive microcircuits, rather than for the moment-to-moment expression of NE-driven synchrony.

This interpretation is consistent with developmental trajectories of prefrontal function, as cognitive flexibility improves during adolescence while NE signaling and prefrontal circuitry mature (Diamond, 2006; Crone and Dahl, 2012; Bradshaw et al., 2016). Nevertheless, our evidence for developmental specificity is indirect, as we did not manipulate *Grin2a* expression at defined stages in LC or mPFC. Lu et al. (2024) reported that conditional deletion of *Grin2a* in adulthood produced minimal behavioral abnormalities, whereas deletion during late postnatal development elicited broad schizophrenia-related phenotypes. Together with our finding that acute GluN2A blockade does not recapitulate constitutive mutant deficits, these results support a model in which GluN2A-dependent maturation is required to establish NE-sensitive LC–mPFC microcircuits. Temporally controlled deletion or rescue experiments will be required to test this model and identify critical windows of vulnerability.

### Clinical and translational implications

Our findings identify the LC→mPFC projection as a GluN2A-dependent circuit that regulates prefrontal synchrony and cognitive flexibility—processes disrupted across multiple neurodevelopmental and psychiatric disorders. In WT slices, NE-induced oscillations required α2-adrenergic receptor activation, nominating α2R-linked mechanisms as a potential therapeutic entry point. Because α2Rs are widely expressed, systemic α2 agonists may produce off-target effects; more targeted strategies (e.g., circuit-focused neuromodulation approaches or subtype-selective α2R modulators) may offer a path to modulate noradrenergic tone with greater specificity.

NE dynamics and LC–mPFC coupling have not yet been directly characterized in GRIN2A mutation carriers, so our findings are best be viewed as a circuit-level hypothesis rather than an established human mechanism. This hypothesis could be tested by assessing LC–prefrontal coupling and NE-dependent modulation of prefrontal activity (e.g., pharmacologic NE challenges combined with EEG or fMRI) in individuals with GRIN2A variants. If GluN2A loss primarily alters circuit maturation, interventions targeting NE–NMDAR interactions may be most effective during sensitive periods, before LC–mPFC disorganization becomes entrenched.

### Limitations and future directions

While our data delineate changes in LC–mPFC function associated with GluN2A deficiency, several constraints warrant consideration. Acute brain slice preparations enable precise circuit interrogation but do not capture state-dependent neuromodulatory dynamics during behavior. In addition, the use of a global Grin2a knock-out prevents localization of the primary locus of dysfunction and does not support an exclusive causal attribution of the behavioral phenotype to the LC–mPFC projection alone.

Future work combining LC and mPFC recordings (or calcium imaging) with cognitive tasks will be important for defining how LC–mPFC interactions evolve across behavioral demands. Region- and cell type–specific deletions of GluN2A in LC, mPFC pyramidal neurons, and prefrontal interneurons will be necessary to disentangle contributions from presynaptic NE release, postsynaptic integration, and top–down cortical feedback. Although we infer the developmental role of GluN2A based on the lack of effect of acute GluN2A blockade and prior developmental versus adult deletion studies, we did not directly test temporally defined LC- or mPFC-specific manipulations. Temporally controlled deletion or rescue in LC and mPFC will be needed to identify critical periods and test causal developmental models. Finally, while we focused on the LC→PrL projection, other circuits also influence reversal learning (Hosseini et al., 2025), underscoring the need to parse contributions from parallel networks converging on mPFC. In particular, measuring NE-induced oscillations and LC–mPFC coupling in adult-conditional *Grin2a* deletion models will help determine whether noradrenergic circuit function remains intact despite minimal behavioral phenotypes reported following adulthood-onset manipulations.

In summary, our findings support the role of GluN2A in shaping LC–mPFC interactions that couple long-range neuromodulation to local prefrontal computations during adaptive cognitive control. Disruption of this coordination provides a circuit-level framework for interpreting cognitive inflexibility associated with GluN2A-linked neurodevelopmental dysfunction.

## Synthesis

Reviewing Editor: Rishikesh Narayanan, Indian Institute of Science Decisions are customarily a result of the Reviewing Editor and the peer reviewers coming together and discussing their recommendations until a consensus is reached. When revisions are invited, a fact-based synthesis statement explaining their decision and outlining what is needed to prepare a revision will be listed below. The following reviewer(s) agreed to reveal their identity: NONE.

Summary of Reviews

In this study, the authors investigate the role of GluN2A in LC-mediated noradrengergic modulation of prefrontal cortex dynamics and cognitive flexibility, using a Grin2a global knockout mouse model. Based on their experiments using optogenetics, electrophysiological, behavioral, and pharmacological techniques, the authors argue that GluN2A is a key determinant of LC-prefrontal circuit function supporting cognitive flexibility, suggesting α2-adrenergic pathways as potential routes to restore prefrontal network coordination.

In the original reviews (transferred through NPRC to eNeuro), the reviewers appreciated the strengths of the paper, the importance of the question that is addressed in the study, and the potential impact of the investigation. However, they expressed critical concerns about causal interpretations that ignored the alternatives, especially given the global knockout of Grin2a. The reviewers provided several alternative interpretations, and underscored the point that global knockouts limit the ability to causally attribute any observed behavioral effects to particular brain circuits, such as the LC-PFC circuit studied here. The reviewers also emphasized the need to account for differences in tasks (between other studies and this one), the lack of data on LC activity or norepinephrine levels in vivo during task performance or on intrinsic properties of PFC neurons, also pointing to several potential compensatory changes that could be triggered by the global knockout.

In this revised submission to eNeuro, the authors have incorporated new data, including acute pharmacology in wild-type slices, anatomical quantification of LC projections, and rigorous validation of optogenetic targeting, along with a comprehensive re-analysis of behavioral and electrophysiological datasets. They provide data showing increased LC fiber density and norepinephrine transporter expression in Grin2a mutants. In their interpretations, they explicitly distinguish between acute signaling roles and the developmental "architectural" role of GluN2A, and interpret this hyperinnervation as compensatory structural sprouting in response to reduced functional integration. They have also rewritten the manuscript to account for several comments from the previous reviews.

Two of the original reviewers have now re-reviewed the revised manuscript. Both reviewers appreciated the new data that has been added to the revised manuscript. However, the reviewers emphasize that the new data underscores the point that the link to the LC-mPFC pathway is weak and indirect. The main concern continues to be about the global nature of Grin2a knockout. Given that NMDA receptors are widely expressed across the brain, it is challenging to causally link behavioral phenotypes to the specific LC-mPFC circuit. The reviewers argue that the data showing that the behavioral phenotype in the Grin2a HET and KO can only be partially affected by LC-mPFC perturbation, and that acute GluN2A pharmacology cannot reproduce the oscillation deficits highlight the challenges associated with such interpretations.

In consensus, the reviewers concur that the results are interesting and valuable, but emphasize the need for a more comprehensive discussion to describe these limitations and the interpretation of the data. For example, the deficits in the Grin2a mice could originate in the LC, anywhere in between LC and mPFC, the mPFC, or other brain regions (developmental adaptation is only one of several possibilities). A revised manuscript should add detailed discussion which clearly incorporates the following:

1. The caveats associated with the causal nature of the interpretations.

2. The several alternate interpretations that the reviewers pointed to in this review and the previous one.

3. Detailed expansion of the compensatory argument that the authors make in their rebuttal.

4. The observation that these results do not support the causal interpretation for a direct link of the observed behavioral effects to the LC-mPFC circuit.

5. The authors must prominently mention the global nature of the knockout and the compensatory argument in their abstract, significance statement, and the introduction of the revised manuscript.

---------------------------------------------------------------------------

Reviews on this revised version submitted to eNeuro

Comments from Original Reviewer #1

Advances the Field (Required)

The authors have now provided convincing evidence the NMDAR-GluN2A is important for locus coeruleus activation that drives cortical rhythms

Comments to the Authors (Required)

The authors have addressed my concerns with additional experiments and revisions of the manuscript

Comments from Original Reviewer #2

Advances the Field (Required)

Interesting data to show the behavioral effects of LC-mPFC perturbation in Grin2a mice, and morphological and electrophysiological quantification of LC neurons in this condition.

Comments to the Authors (Required)

This is a revision of a manuscript originally submitted to a different journal. The authors have made considerable amount of edits, and the data are interesting. However, the link to the LC-mPFC pathway is weak and indirect.

The main concern is still about that NMDA receptors are widely expressed across the brain, and it is challenging to pinpoint to the specific LC-mPFC circuit. In fact, the data showing that the behavioral phenotype in the Grin2a HET and KO can only be partially affected by LC-mPFC perturbation, and that acute GluN2A pharmacology cannot reproduce the oscillation deficits are in support of this view. A more comprehensive discussion is needed to describe these limitations and the interpretation of the data. For example, the deficits in the Grin2a mice could originate in the LC, anywhere in between LC and mPFC, the mPFC, or other brain regions (developmental adaptation is only one of several possibilities). The current results, albeit interesting and valuable, do not support a direct link to the LC-mPFC circuit, in my opinion.

---------------------------------------------------------------------------

Original reviews received through NPRC

Comments from Reviewer #1

Comments to the Author:

This paper has several strengths. It shows that locus coeruleus (LC) modulation of the medial prefrontal cortex (mPFC) in mice has a beneficial effect on cognitive flexibility, irrespective of the role of NMDAR- GluN2A expression. It also shows that knockdown of GRIN2A impairs this cognitive flexibility. What is less clear is that the lack of significant effect of LC manipulations in Grin2aKO animals is due to the loss of NMDAR-GluN2A specifically in LC neurons.

The mPFC in rodents (J Neurosci. 2003 Jul 2;23(13):5472-6; Learn Mem. 20: 290-4, 2013), and the dorsolateral PFC in primates (Neuron. 2013 Feb 20;77(4):736-49), rely on NMDAR-GluN2A for their normal functioning, with neurotransmission in dlPFC almost fully dependent on NMDAR actions. Furthermore,

the PFC projects down to the LC and is important for regulating its activity (Brain Res. 1984 Jul 23;306(1-

2):9-18; Proc Natl Acad Sci U S A. 1995 Jun 20;92(13):6032-6; Neuroscience. 1998 Mar;83(1):63-79.). An alternative interpretation of their data is that noradrenergic neuromodulation of mPFC synapses cannot have any effect if there is no or limited neurotransmission in the mPFC to modulate. A strict test of their hypothesis would require selective removal of GRIN2A limited to LC neurons.

Figure 1 panels E and F are the heart of the paper and yet the data points are so close together that it is hard to see changes with stimulation. It is clear that LC excitation improved WT and in het animals, while inhibition impaired WT performance. However, both inhibition and excitation seem to have had mixed effects in the Grin2aKO animals, where it appears that those animals with good performance at baseline were worsened by LC stimulation (either excitation or inhibition of LC), and those with baseline poor performance were improved by either treatment (Figs. 1E,F). This is called "regression to the mean". This pattern also seems evident for the het animals with LC inhibition. This pattern would also fit with an inverted U dose response in NE actions. This alternative interpretation would respect the finding that excessive levels of NE can impair mPFC function.

It is of interest that a previous study has shown that NMDA blockade within the LC increased NE release in the orbital cortex (Biomolecules. 2020 Jul 1;10(7):990). These data suggest that there are also NMDA receptors on GABA interneurons in the LC and thus that Grin2aKO might disinhibit LC and increase firing? The current study found that Grin2aKO neurons had reduced current amplitude (Fig. S3F), but they did

not record from the LC in vivo and thus we do not know if firing rates would be different eg during task performance.

The oscillation data show that NE increases oscillations via alpha-2-adrenoceptors, but the relevance to Grin2a actions on LC neurons is not clear, as there are ceiling effects at baseline in these animals. This may be due to the increased NE innervation of the mPFC in response to deletion of Grin2a, but that hyperinnervation could be a compensation for reduced activity in the mPFC rather than inherent changes in LC neurons.

There are many errors in the text which erode confidence in the study, e.g.:

The legend for Figure 5 does not fit- the figure is about NE antagonists, not a GluN2A antagonist. The legend for Figure S3 does not explain panels D and E

The title of Figure S4 is "Yohimbine Reverses NE-Induced Oscillations in WT mPFC" and yet there are no

yohimbine data in the figure

There are missing decimal points in the Tables

Comments from Reviewer #2

Comments to the Author:

In the present study, Hosseini et al. investigated the role of GluN2A in LC-mediated modulation of prefrontal cortex dynamics and cognitive flexibility, using a Grin2a knockout mouse model. They observed bidirectional modulation of reversal learning by optogenetic perturbations of LC→mPFC circuit in WT mice, but not in Grin2a mutants. Grin2a mutants exhibited impaired reversal learning, disrupted oscillations in the mPFC. Furthermore, optogenetic stimulation of LC terminals as well as exogenous norepinephrine failed to induce oscillatory activity in mPFC of Grin2a mutants. They show that α2- adrenoceptors were required for NE-driven cortical dynamics.

This work presents potentially impactful investigation about the crucial role of GluN2A as essential for LC- PFC network. However, several conceptual and experimental concerns limit the interpretability and impact of the findings in the current form.

I provide my detailed comments below:

It is not clear from the methods whether the Grin2a knockout is global or conditional. This is a crucial point, as a global knockout would substantially limit the ability to attribute any observed behavioral or neurotheological effects to particular brain circuits, such as LC-PFC studied here. It is also quite possible that reversal learning is not the only behavioral paradigm that are impaired in the mutants. Further evidence is needed to substantiate the specificity and direct causality linking Grin2a to LC-PFC in reversal learning.

The authors observed impaired reversal learning and disrupted oscillatory activity in Grin2a mutants but are unable to rescue these deficits by perturbing LC input. This weakens the causal interpretation of GluN2A's role in the LC-PFC pathway. Are intrinsic properties of mPFC neurons disrupted in the mutants as well? This brings us back to the previous comment that, if Grin2a KO is global, many different factors can affect reversal learning and other behavior through multiple circuits.

Several experimental sections are not conclusive. To our knowledge, α2 receptors are expressed both presynaptically and postsynaptically. How to conclude that exogenous application of NE to mPFC slices affects postsynaptic compartments? In addition, GluN2A blockade cannot replicate the oscillatory deficits, to the reviewer, it suggests that the observed effects are just correlational, but not causal, raising further concerns of investigating the LC-PFC circuit in this context.

A critical experiment to examine the abnormality of the LC-PFC circuit is to directly measure NE levels (e.g., via microdialysis or fiber photometry) in response to LC stimulation in WT vs. mutants. Stimulating LC with simultaneous a2 pharmacology should provide additional insights.

Fig.1C shows optic fibers placed at a distance from LC neurons expressing opsins. This raises concerns about effective light delivery. The authors should provide detailed histological validation for all mice used in optogenetic experiments.

The number of LC neurons projecting to the mPFC should be quantified across all genotypes to rule out developmental connectivity differences.

The authors should test lower frequency of optic stimulations of LC neurons, e.g. below 15 Hz in addition to 25 Hz to see if it has effect on behavior or mPFC activity.

Comments from Reviewer #3

Comments to the Author:

The study by Hosseini et al. determines how Grin2a ablation disrupts noradrengergic modulation of the

PFC and cognitive flexibility. They found that mutations in GluN2a receptor expression disrupted cognitive

flexibility. Moreover, these deficits were recapitulated by optogenetic inhibition of the LC-to-mPFC circuit, whereas activation of the same circuit improved performance in wild-type and heterogeneous mice. The authors also found that cortical gamma oscillations were altered in mutant mice. While the study addresses an important question, the manuscript would benefit from substantial revisions to improve clarity, methodological transparency, and overall organization. The comments below are offered in the spirit of strengthening the work.

The authors suggest that discrepancies in the literature regarding reversal learning deficits in Grin2a mutant mice may be due to differences in task design or methodology. However, the two cited studies used an attentional set-shifting task and a touchscreen-based visual discrimination/reversal learning task, respectively, both of which differ considerably from the T-maze reversal learning task employed in the present study. This should be more clearly acknowledged, and the relevance of the cited studies to the current work should be justified or reconsidered.

The methods section lacks a detailed description of how behavioral data were analyzed. While Figure 1 indicates a paired t-test was used, these data may be more appropriately analyzed using ANOVA, particularly if multiple conditions or phases are being compared. Additionally, the specific comparison being made is not clearly stated-presumably, this refers to performance in the initial reversal phase (without stimulation) compared to the subsequent reversal phase (with stimulation), but this should be explicitly clarified.

Regarding the optogenetic stimulation parameters, the manuscript states that stimulation was delivered for 10 seconds when the animal was in the start box, whereas trial duration ranged from 1-3 minutes. It remains unclear whether the stimulation encompassed the entire trial or whether the trial commenced only after the stimulation period had elapsed. Clarification on the temporal alignment of stimulation relative to behavioral performance is essential.

The description of the training protocol notes that mice selected a "pseudorandomized" goal arm until reaching criterion. It is unclear whether the goal arm was randomized on a trial-by-trial basis or counterbalanced across mice. The latter seems more likely, but clarification is necessary. This is particularly important given that optogenetic stimulation was unilaterally targeted to the right hemisphere. Was any consideration given to potential lateralization effects, i.e., whether the side of the goal arm interacted with performance during optogenetic stimulation sessions?

Several sections of the manuscript would benefit from more careful editing. Some paragraphs lack coherence or end abruptly, and there are instances where content appears tangential or redundant. For example, the penultimate paragraph of the Introduction discusses dopaminergic (DA) disruptions, yet DA is not directly manipulated or assessed in this study. Similarly, the optogenetic stimulation protocol is described multiple times across different sections (e.g., Behavioral Setup and Training vs. Optogenetic Intervention), and technical details such as optic fiber specifications are repeated unnecessarily. The first section of the Results ends with the sentence: "These findings confirm that Grin2a mutants exhibit spatial

reversal-learning deficits and suggest broader impairments in adaptive behavior." This sentence reiterates earlier points without clearly summarizing the data presented in that section-namely, modulation of LC activity and its effect on performance. There is also an apparent mismatch between the figure cited and the data discussed. The improvement in reversal learning performance following optogenetic activation of the LC-mPFC pathway is linked to Fig. S1; however, this figure does not appear to include behavioral data. The authors should ensure accurate figure referencing throughout.

The Discussion section asserts that LC-NE circuit dysfunction underlies the observed cognitive impairments. However, the electrophysiological findings suggest that the primary deficit may be postsynaptic. Given the broad expression of GluN2A receptors, it is conceivable that the LC-mPFC circuit is not specifically disrupted in Grin2a mutants. Instead, impaired function of mPFC neurons (e.g., pyramidal or GABAergic cells) may contribute more directly to the reversal learning deficits. This alternative explanation should be discussed.

Minor comments

The rationale for using ChR2 in electrophysiological experiments and ChETA in behavioral studies should be clarified.

Please confirm whether the reward size was truly 1 mg or if this is a typographical error.

While 110 nl is specified for the electrophysiology experiments, the viral volumes used for behavioral injections into the mPFC and LC should also be reported.

The LC contains heterogeneous populations, including serotonergic neurons. What proportion of TH- positive neurons co-expressed eYFP/fusionRed, and conversely, what percentage of labeled neurons were TH-positive?

---------------------------------------------------------------------------

## Author Response

June 9, 2026 Rishikesh Narayanan, PhD Reviewing Editor, eNeuro Re: Revised manuscript eN-NWR-0050-26, "GluN2A Enables Noradrenergic Control of Prefrontal Oscillations and Cognitive Flexibility" Dear Dr. Narayanan, Thank you for the careful synthesis of the reviews and for the opportunity to revise our manuscript. We appreciate the reviewers' recognition that the new anatomical, electrophysiological, pharmacological, behavioral, and targeting-validation analyses strengthen the study. In this revision, we focused on the central interpretive concern raised in the synthesis: because the Grin2a model is a global knockout and GluN2A is broadly expressed, the present data cannot assign the behavioral phenotype exclusively or causally to the LC-mPFC projection.

We revised the manuscript to make this limitation prominent from the front matter through the Discussion. The Abstract, Significance Statement, and Introduction now identify the global nature of the model and frame the observed noradrenergic hyperinnervation as compensatory structural remodeling that may reflect developmental adaptation. The Results and Discussion now distinguish the projection-specific findings from broader interpretations of the mutant phenotype, discuss alternate loci of dysfunction, expand the compensatory argument, and state explicitly in the Limitations section that the global model does not support an exclusive causal attribution of the behavioral phenotype to the LC-mPFC projection alone.

Below we provide a point-by-point response. Reviewer and editor comments are paraphrased for clarity, followed by our responses.

Response to Manuscript Instruction Comment Please revise the Extended Data naming convention so that each Extended Data item is named after the figure or table it extends.

Response We revised the Extended Data labels to follow eNeuro figure-based naming. The revised manuscript now lists Extended Data items as figure extensions such as Figure 1-1, Figure 1-2, Figure 1-3, Figure 2-1, Figure 2-2, Figure 2-3, Figure 2-4, Figure 3-1, and Figure 4-1, and the title-page count has been updated accordingly.

Response to Synthesis Statement Comment 1 The revised manuscript should describe the caveats associated with causal interpretation, especially given the global Grin2a knockout.

Response We agree and have revised the manuscript throughout to avoid attributing the behavioral phenotype exclusively to the LC-mPFC projection. The Abstract now identifies the knockout as global. The Introduction explains that observed behavioral deficits may reflect systemic contributions or developmental adaptations across multiple brain regions. In the Results, after reporting the behavioral effects of LC-mPFC manipulation, we now state that the global model does not distinguish among impaired mPFC responsiveness, altered LC function, or broader developmental adaptations outside this projection. In the Discussion and Limitations section, we explicitly state that the global knockout prevents localization of the primary locus of dysfunction and does not support an exclusive causal attribution of the behavioral phenotype to the LC-mPFC projection alone.

Comment 2 The Discussion should incorporate alternate interpretations raised in the current and prior reviews.

Response We expanded the Discussion to incorporate these alternatives. The revised text now discusses that reduced behavioral sensitivity to LC-mPFC manipulation in Grin2a mutants may arise from impaired integration of noradrenergic signals within prefrontal networks, altered LC physiology or feedback, developmental changes in other circuits supporting reversal learning, or a combination of these mechanisms. We also discuss the possibility that the oscillatory phenotype reflects disruption of alpha2-adrenergic microcircuits and/or downstream cortical integration rather than a simple failure of LC output. Finally, the Limitations and future directions section now identifies region- and cell type-specific deletion or rescue experiments in LC, mPFC pyramidal neurons, and prefrontal interneurons as necessary tests of these alternatives.

Comment 3 The compensatory argument should be expanded in detail.

Response We expanded this argument in the Abstract, Introduction, Results, and Discussion. The revised manuscript now states that Grin2a mutants show increased LC axonal density and elevated norepinephrine transporter expression in prelimbic cortex and frames these structural findings as noradrenergic remodeling rather than simple loss of LC input. In the Discussion, we explain that increased LC axonal density and NET expression may represent compensatory reorganization in response to reduced functional integration of LC-derived NE signals within mPFC networks. We also state that these anatomical and molecular changes do not establish the direction of extracellular NE change during behavior and could affect release, clearance, or temporal precision of neuromodulatory signaling.

Comment 4 The manuscript should clearly acknowledge that the present results do not support a direct causal interpretation linking the observed behavioral effects to the LC-mPFC circuit.

Response We have made this point explicit. The revised Limitations section states that the global Grin2a knockout prevents localization of the primary locus of dysfunction and "does not support an exclusive causal attribution of the behavioral phenotype to the LC-mPFC projection alone." We also revised the summary of the findings to describe LC-mPFC signaling as one neuromodulatory pathway altered in the setting of global GluN2A deficiency, rather than as the sole causal origin of the mutant behavioral phenotype.

Comment 5 The global nature of the knockout and the compensatory argument should be prominently mentioned in the Abstract, Significance Statement, and Introduction.

Response We addressed this requirement in all three front-matter sections. The Abstract now specifies that the optogenetic behavioral findings were obtained in global Grin2a knockouts and states that the functional deficits should be interpreted in the context of compensatory structural hyperinnervation that may reflect developmental adaptation rather than acute signaling loss. The Significance Statement now emphasizes global GluN2A deficiency, potential systemic and developmental contributions, and compensatory changes in circuit architecture. The Introduction now includes a paragraph explaining that the global model can reveal chronic structural reorganization while also limiting localization of the behavioral deficit to a single pathway.

Responses to Reviews on the Revised Version Original Reviewer 1 Comment The additional experiments and revisions address the reviewer's concerns.

Response We thank the reviewer for the positive assessment and for the earlier comments that helped improve the rigor and interpretive precision of the manuscript.

Original Reviewer 2 Comment 1 The link to the LC-mPFC pathway remains weak and indirect because NMDA receptors are broadly expressed and the global knockout limits circuit-specific causal inference.

Response We agree that the global knockout limits circuit-specific causal inference. We revised the Abstract, Significance Statement, Introduction, Results, Discussion, and Limitations to make this point prominent. The revised manuscript now separates the direct findings, namely that LC-mPFC manipulation modulates reversal learning in wild-type mice and that LC terminal stimulation or norepinephrine application recruits oscillatory activity most reliably in wild-type mPFC slices, from the broader interpretation of the Grin2a mutant phenotype. We now state explicitly that the mutant behavioral phenotype cannot be assigned exclusively to the LC-mPFC projection using the present model.

Comment 2 The partial behavioral effects of LC-mPFC perturbation in Grin2a HET and KO mice and the inability of acute GluN2A pharmacology to reproduce the oscillation deficits should be incorporated into the interpretation.

Response We incorporated both observations into the revised Results and Discussion. The behavioral Results now note that activation effects are partially preserved in Grin2a HET mice and absent in Grin2a KO mice, that inhibitory effects are blunted in mutants, and that the global model cannot distinguish among altered mPFC responsiveness, altered LC function, or broader developmental effects. The Discussion now treats the failure of acute NVP-AAM077 treatment to reproduce the constitutive mutant oscillation phenotype as evidence that the phenotype may reflect long-term circuit maturation or developmental adaptation rather than an acute requirement for GluN2A during NE-driven oscillation induction.

Comment 3 A more comprehensive Discussion should consider that deficits could arise in LC, in mPFC, between the two structures, or in other brain regions; developmental adaptation is only one possibility.

Response We expanded the Discussion accordingly. The revised manuscript now discusses altered LC physiology and cortical feedback, impaired mPFC integration of NE signals, alpha2-adrenergic microcircuit or downstream integration failures, developmental changes in other circuits that support reversal learning, and parallel networks converging on mPFC. We also clarify that our developmental interpretation is indirect and requires temporally controlled and region-specific manipulations to test.

Comment 4 The current results do not support a direct link between the observed behavioral phenotype and the LC-mPFC circuit.

Response We agree that the present global model does not establish an exclusive direct causal link for the behavioral phenotype. We revised the manuscript to state this explicitly in the Limitations section and tempered summary language elsewhere. The revised framing is that LC-mPFC signaling is altered in the setting of global GluN2A deficiency and is one pathway relevant to the observed behavioral and network phenotypes, while additional circuit-specific experiments are required to localize causality.

Summary of Principal Revisions 1. The Abstract, Significance Statement, and Introduction now prominently identify the global Grin2a model and the compensatory structural interpretation.

2. The Results now temper the behavioral interpretation of LC-mPFC manipulations in mutants and acknowledge regression toward the mean when interpreting individual apparent improvements from poorer baseline performance.

3. The Discussion now expands alternate interpretations involving LC physiology, mPFC integration, reciprocal feedback, alpha2-adrenergic microcircuits, developmental adaptation, and parallel circuits.

4. The Discussion now expands the compensatory interpretation of increased LC axonal density and NET expression while acknowledging that extracellular NE dynamics during behavior were not directly measured.

5. The Limitations section now explicitly states that the global knockout prevents localization of the primary dysfunction and does not support an exclusive causal attribution of the behavioral phenotype to LC-mPFC alone.

6. Extended Data labels were revised to match eNeuro figure-based naming.

We thank you and the reviewers again for the constructive feedback. We believe these revisions make the scope of the conclusions clearer while preserving the central observation that GluN2A deficiency is associated with altered noradrenergic regulation of prefrontal network dynamics and cognitive flexibility.
