# Supplementary material for: GluN2A Enables Noradrenergic Control of Prefrontal Oscillations and Cognitive Flexibility
Source: eNeuro. 2026 Aug 4;13(8):ENEURO.0050-26.2026. doi: 10.1523/ENEURO.0050-26.2026 (PMC13456957; doi:10.1523/ENEURO.0050-26.2026)
Supplement: Supplementary file 1 [file eneuro-13-ENEURO.0050-26.2026-s008.docx]

|  | | **Layer II / III** | | **Layer V** | |
| --- | --- | --- | --- | --- | --- |
| Oscillation Type | Genotype | Baseline  (Mean ± SEM) | Stimulated  (Mean ± SEM) | Baseline  (Mean ± SEM) | Stimulated  (Mean ± SEM) |
| **iHFO** | *Grin2a*^WT^ | 0.509 ± 0.036 | ***0.761 ± 0.066*** | 0.560 ± 0.028 | ***0.885 ± 0.079*** |
|  | *Grin2a*^HET^ | 0.549 ± 0.053 | 0.665 ± 0.103 | 0.622 ± 0.044 | 0.709 ± 0.099 |
|  | *Grin2a*^KO^ | 0.724 ± 0.063 | 0.759 ± 0.062 | 0.759 ± 0.067 | 0.793 ± 0.085 |
| **iGBO** | *Grin2a*^WT^ | 0.465 ± 0.050 | ***0.889 ± 0.117*** | 0.556 ± 0.036 | ***1.038 ± 0.138*** |
|  | *Grin2a*^HET^ | 0.573 ± 0.102 | 0.738 ± 0.173 | 0.605 ± 0.061 | 0.726 ± 0.109 |
|  | *Grin2a*^KO^ | 0.771 ± 0.106 | 0.833 ± 0.108 | 0.805 ± 0.102 | 0.858 ± 0.137 |

**Figure 3-1. Mixed-effects analysis of optogenetically induced oscillatory power following LC terminal stimulation in mPFC slices.**

Results of linear mixed-effects models assessing changes in oscillatory power evoked by optogenetic stimulation of LC terminals in mPFC slices from WT, *Grin2a*^HET^, and *Grin2a*KO mice. Slice identity was included as a random intercept, with fixed effects of genotype, stimulation condition (baseline vs stimulation), and genotype × condition interaction. Reported Δ values reflect Tukey-adjusted within-genotype contrasts (stimulation − baseline) with 95% confidence intervals and multiplicity-adjusted p values. Δ values are reported in mV².
