# Supplementary material for: GluN2A Enables Noradrenergic Control of Prefrontal Oscillations and Cognitive Flexibility
Source: eNeuro. 2026 Aug 4;13(8):ENEURO.0050-26.2026. doi: 10.1523/ENEURO.0050-26.2026 (PMC13456957; doi:10.1523/ENEURO.0050-26.2026)
Supplement: Supplementary file 2 [file eneuro-13-ENEURO.0050-26.2026-s009.docx]

|  | | **Layer II / III** | | **Layer V** | |
| --- | --- | --- | --- | --- | --- |
| **Oscillation Type** | **Genotype** | **Baseline**  (Mean ± SEM) | **NE-Evoked**  (Mean ± SEM) | **Baseline**  (Mean ± SEM) | **NE-Evoked**  (Mean ± SEM) |
| **HFO** | *Grin2a*^WT^ | 0.395 ± 0.025 | ***0.693 ± 0.107*** | 0.505 ± 0.053 | ***1.009 ± 0.072*** |
|  | *Grin2a*^HET^ | 0.575 ± 0.122 | 0.660 ± 0.112 | 0.578 ± 0.081 | 0.818 ± 0.105 |
|  | *Grin2a*^KO^ | 0.717 ± 0.054 | 0.920 ± 0.076 | 0.512 ± 0.066 | 0.795 ± 0.126 |
| **GBO** | *Grin2a*^WT^ | 0.415 ± 0.028 | ***0.755 ± 0.119*** | 0.541 ± 0.061 | ***1.014 ± 0.089*** |
|  | *Grin2a*^HET^ | 0.523 ± 0.121 | 0.658 ± 0.119 | 0.561 ± 0.091 | 0.813 ± 0.124 |
|  | *Grin2a*^KO^ | 0.719 ± 0.064 | 1.019 ± 0.136 | 0.491 ± 0.072 | 0.782 ± 0.133 |

**Figure 4-1. Mixed-effects analysis of norepinephrine-evoked oscillatory power in mPFC slices.**

Results of linear mixed-effects models assessing changes in oscillatory power evoked by bath application of norepinephrine (NE) in mPFC slices from WT, *Grin2a* HET, and *Grin2a* KO mice. Slice identity was included as a random intercept, with fixed effects of genotype, condition (baseline vs NE), and genotype × condition interaction. Reported Δ values reflect Tukey-adjusted within-genotype contrasts (NE − baseline) with 95% confidence intervals and multiplicity-adjusted p values. Δ values are reported in mV² to match descriptive units.
